# Supplementary material for: Human miRNA miR-675 inhibits DUX4 expression and may be exploited as a potential treatment for Facioscapulohumeral muscular dystrophy
Source: Nat Commun. 2021 Dec 8;12:7128. doi: 10.1038/s41467-021-27430-1 (PMC8654987; doi:10.1038/s41467-021-27430-1)
Supplement: Supplementary file 1 — Supplementary Information [file 41467_2021_27430_MOESM1_ESM.pdf]

Human miRNA *miR-675* inhibits *DUX4* expression and may be exploited as a potential treatment for Facioscapulohumeral muscular dystrophy

Nizar Y. Saad[1], Mustafa Al-Kharsan[1,2], Sara E. Garwick-Coppens[1], Gholamhossein Amini Chermahini[1], Madison A. Harper[1], Andrew Palo[1], Ryan L. Boudreau[3] and Scott Q. Harper[1,4*]

Supplementary Note 1. Bioinformatics and reporter assay using the miRbase algorithm.

To investigate the hypothesis that *DUX4* was regulated by endogenous miRNAs, we first used the miRNA target prediction algorithm miRBase to identify miRNAs potentially targeting *DUX4* sequences (1).

When we began this work, conventional thinking suggested that miRNAs almost exclusively target 3'UTR regions, and we thus used miRBase to predict miRNAs targeted to the *DUX4* 3'UTR, as well as potentially retained introns. However, we also hypothesized that miRNAs might help suppress full-length *DUX4* and favor production of the alternative, non-toxic *DUX4*-short (*DUX4-s*) splice form, and thus also identified a subset that were predicted to bind the 3' end of the full-length *DUX4* open reading frame (ORF) (Supplementary Fig. 1). We cloned twenty-four miRNAs with high miRBase prediction scores into expression plasmids containing the U6 promoter and then performed *in vitro* gene silencing testing using a *DUX4*-customized dual luciferase assay. In addition to the U6 promoter-driven miRNA expression constructs (2), this experiment required luciferase reporter plasmids containing *DUX4* or positive control sequences. Specifically, using the commercial dual luciferase plasmid psiCheck2 (Promega), we cloned the *DUX4* open reading frame (ORF) and 3' UTR/intronic regions (RenLuc-DUX4 ORF and RenLuc-DUX4 3'UTR, respectively) downstream of the *Renilla* luciferase stop codon, such that *DUX4* sequences operated as the 3'UTR of *Renilla* luciferase. In this configuration, a fusion RNA, but not fusion protein, was created. In addition, we also generated a positive control luciferase construct containing perfect target sites of all 23 miRBase-predicted miRNAs (RenLuc-PTS, where PTS =

perfect target sites). Each luciferase plasmid also contained a separate *Firefly* luciferase cassette that was used as a normalizer of the luminescent signal (Supplementary Fig. 1). To validate the assay, we used a designed *DUX4*-targeting miRNA (*miDUX4.405*), which binds a cognate sequence in the *DUX4 ORF*, as both a positive and negative control (3). We co-transfected the U6.miRNA and luciferase reporter plasmids in HEK293 cells and measured *Renilla/Firefly* luciferase activity 24 hours later. As expected, the *miDUX4.405* positive control reduced *RenLuc-DUX4 ORF* expression by ~80% but did not significantly reduce *RenLuc-DUX4 3' UTR* expression, as the *DUX4* 3'UTR does not contain a *miDUX4.405* binding site (Supplementary Fig. 1). 7 out of the 23 miRBase-predicted constructs mediated poor *DUX4* gene silencing of the *DUX4 ORF* construct in this assay. All predicted miRNAs functioned to silence their perfect target sites, suggesting that they were expressed and processed to a functional form (Supplementary Fig. 1a-d).

Supplementary Note 2. *MiR-675-5p*, but not *miR-675-3p*, regulates DUX4.

A previous report showed that two functional *miR-675* mature sequences (*miR-675-3p* and *-5p*) are processed from the *H19* long non-coding RNA (4). To confirm this, we transfected the human *H19* construct (CMV.H19) into HEK293 cells and used QPCR assays specific for mature processed miRNA sequences, 24h after transfection. As previously reported, we found that both *miR-675-3p* and *miR-675-5p* were processed as mature miRNAs from the primary *H19/miR-675* transcript, with mature *miR-675-3p* being 2.20±0.15-fold more abundant than *miR-675-5p* in this assay (P<0.0001, ANOVA, N=3) (Supplementary Fig. 2a). Since the PITA algorithm only predicted *DUX4* regulation by the *miR-675-5p* strand, we were primarily concerned with the -5p species, but because both mature miRNAs were produced from the same primary transcript, we wanted to determine if *miR-675-3p* was capable of silencing *DUX4* or not. To do this, we generated *miR-675* expression constructs designed to specifically produce only *miR-675-5p* or *miR-675-3p* and repeated luciferase assay experiments using the DUX4-FL target. This

experiment confirmed that *miR-675-5p* reduced *Renilla* luciferase activity from the DUX4-FL construct by 33±3% ($P<0.0001$, ANOVA, N=3), while the *miR-675-3p* sequences had no significant impact (Supplementary Fig. 2b).

Supplementary Note 3. Confirmation of U6.MIR675 silencing of a perfect target site.

Typically, greater complementarity between inhibitory RNAs and their target sites promotes more robust silencing. Indeed, our artificial therapeutic microRNAs contain perfect 22 nt complementarity with target genes to facilitate maximum gene silencing. With this principle in mind, we therefore generated a *Renilla* luciferase construct containing a perfect target site for the mature *miR-675-5p* sequence (miR-675R*, where* R=reverse complement), to help determine the maximum knockdown we could achieve with the U6.MIR675 expression plasmid. Using this target, we were surprised to find that the U6.MIR675 plasmid only achieved 41±12% ($P=0.01$, ANOVA, N=3) silencing in the luciferase assay at the highest ratios of miRNA to reporter plasmid (12:1), in contrast to the >80% knockdown we typically see with the *miDUX4.405* positive control (Supplementary Fig. 2d). We hypothesized that mature *miR-675-5p* was not optimally expressed and/or processed from the U6.MIR675 plasmid, and therefore tested another *miR-675* expression plasmid utilizing the H1 promoter (H1.MIR675) (Fig. 1c).

Supplementary Note 4. *MiR-675* reduces *DUX4* mRNA *in vitro*.

RNA interference can occur through two mechanisms: translational inhibition and target mRNA degradation. To determine if *miR-675* impacted *DUX4* mRNA stability, we next measured *DUX4* mRNA levels following co-transfection of CMV.DUX4-FL/CMV.eGFP with H1.MIR675 or the U6.miLacZ negative control plasmids in HEK293 cells. To do this, we extracted RNA 24 or 48h after transfection and measured absolute and relative *DUX4* mRNA expression using droplet digital PCR (ddPCR) (*DUX4* mRNA absolute concentration in copies/μL) and real-time quantitative PCR (relative *DUX4* expression), respectively. In the ddPCR experiment, H1.MIR675

significantly reduced *DUX4* mRNA levels by 71±2% (P<0.0001, ANOVA, N=3) compared to the miLacZ negative control, 48h after transfection (Fig. 1d). In the QPCR experiment, H1.MIR675 also significantly reduced *DUX4* mRNA levels by 37±2% (P<0.0001, ANOVA, N=3), 24h after transfection, and by 51±2% (P<0.0001, ANOVA, N=3), 48h after transfection (Supplementary Fig. 9a). These results suggested that *miR-675* could mediate degradation of *DUX4* mRNA, although additional silencing by translational inhibition could not be ruled out.

Supplementary Note 5. Validating anti-miR-675 antagomir functionality and measuring *miR-675* and *H19* expression in FSHD myoblasts.

Prior to using these reagents experimentally, we opted to first validate their functionality by confirming *miR-675* and *DUX4* gene expression in 15A, 17A, and 15V cells, and empirically testing the impact of inhibiting *miR-675* with an antagomir on known *miR-675* targets. First, to validate the cell lines, we confirmed the expression patterns of endogenous *DUX4* and *miR-675* expression in 15A, 17A and 15V cell lines using QPCR. Consistent with published work, we detected *DUX4* in 4-day differentiated (4DD) 15A and 17A myotubes, but not in 15V cells treated under the same differentiation conditions (Supplementary Fig. 10). Prior studies suggested these cells should express *miR-675* and *H19*, which are predominantly embryonic genes that become downregulated after birth in all tissues except differentiated skeletal muscles, but their levels had not yet been measured in 15A, 17A and 15V cells (4). We therefore examined *H19* and *miR-675* expression under growth (myoblast) and differentiation (myotubes) conditions using QPCR to detect *H19*, *pri-mir-675*, and the individual mature *miR-675* strands (*miR-675-5p* and *miR-675-3p*, where the -5p strand is *DUX4* targeting). All 3 cell lines expressed the various *H19*/*miR-675* species in both the myoblast and myotube stages, and expression increased upon differentiation (Supplementary Table 1 and Supplementary Fig. 10). *H19* expression was more pronounced than that of *miR-675* in myotubes. *H19* has other known functions as a full-length non-coding RNA beyond serving as a precursor of *miR-675*, and previous studies suggested processing of

H19 to *miR-675* is inefficient (5). The differences in expression level between *H19* and *miR-675* suggest the majority of *H19* remains unprocessed.

Next, we assessed the functionality of an *anti-miR-675* antagomir to inhibit *miR-675-5p* in human myoblasts. A prior study showed that *miR-675-5p* inhibited the DNA replication initiation factor *CDC6*, and we confirmed this regulation in HEK293 cells (Supplementary Fig. 2c). We then used endogenous CDC6 protein expression in human myotubes as a readout of antagomir functionality (4). Since *miR-675* was a known *CDC6* inhibitor, we expected that inhibiting the inhibitor (i.e. silencing *miR-675-5p* with *anti-miR-675* antagomir) would de-repress *CDC6*, thereby increasing CDC6 protein expression. To test this, we transfected 15V cells with either a scrambled control (anti-miR -ctrl) or the *anti-miR-675* antagomir, differentiated cells for 4 days, and then performed western blotting. We found CDC6 protein expression only in myotubes that received the *anti-miR-675* antagomir, but no detectable signal in myotubes that received the *anti-mir-ctrl* (Supplementary Fig. 11a). We also performed a similar experiment in both 15V and 15A cells, using a dual luciferase assay in which *Renilla* luciferase contained a perfect *miR-675-5p* target site (RenLuc-miR-675R). Again, the *anti-miR-675* antagomir de-repressed relative *Renilla* luciferase activity in both cell lines, while the negative control antagomir did not (Supplementary Fig. 11b). Thus, together these studies supported that the antagomir system was appropriate for assessing endogenous *miR-675* regulation of *DUX4* sequences in 15A, 17A and 15V muscle cells.

Supplementary Note 6. Inhibition of *miR-675* increases cell death in FSHD cells.

To extend these *miR-675* inhibition studies, we also found that antagomir-mediated knock down of *miR-675-5p* in 15A myotubes - not transfected with *DUX4* - provoked a 1.8±0.1-fold ($P<0.0001$, ANOVA, N=3) increase in Caspase 3/7 activity (Fig. 7a). As a positive control, the transfection of DUX4-FL and DUX4-miR-675Res expression plasmids provoked a larger increase in Caspase 3/7 activity of 5.6±0.4-fold ($P<0.0001$, ANOVA, N=3) and 10.6±0.8-fold ($P<0.0001$, ANOVA, N=3),

respectively, supporting a DUX4-dependent response that is affected by *miR-675*. These results suggested that endogenous *miR-675* may help check DUX4-induced toxicity in FSHD human muscle cells.

Supplementary Note 7. Validation of *miR-675* drug therapies in transfected HEK293 cells.

We treated cells with each small molecule (10 and 20 μM β-Estradiol and MPA or 20 μM β-Estradiol alone; 20 and 40 μM melatonin) and then measured *miR-675-5p* expression by ddPCR 24 hours later. We found that the three treatment regimens, β-estradiol, β-estradiol + MPA and melatonin, significantly increased *miR-675* levels compared to mock-treated (ethanol), *DUX4*-transfected cells (Supplementary Data 5 and Supplementary Fig. 18). In addition, 20 μM β-estradiol, β-estradiol + MPA (20 μM each) or melatonin (20 and 40 μM) significantly decreased levels of *DUX4* and the *DUX4*-responsive biomarker *TRIM43* (Supplementary Data 5). Co-transfection with *anti-miR-675* increased *TRIM43* levels when HEK293 cells were treated with 10 μM of the combination β-estradiol + MPA and 20 μM of melatonin, indicating that the drugs exerted their effect on *DUX4* and *TRIM43* by directly inducing the expression of *miR-675*. Finally, *TRIM43* expression did not decrease in melatonin-treated HEK293 cells transfected with the *miR-675*-resistant *DUX4* plasmid, supporting that the effects of melatonin on *DUX4* were *miR-675*-dependent (Supplementary Data 5 and Supplementary Fig. 18).

Supplementary Note 8. Potential direct effects of β-estradiol, β-estradiol+MPA, or melatonin on *TRIM43* levels.

Our data in Fig. 9 supported the hypothesis that RNAi mediated by small molecule upregulation of *miR-675* could reduce *DUX4* and indirectly decrease a DUX4-responsive biomarker in FSHD muscle cells. Nevertheless, as a control, we further considered the possibility that the small molecules could directly reduce *TRIM43* levels. The most straightforward method to address this possibility was to measure *TRIM43* in *DUX4*-negative, unaffected myotubes treated with each

molecule (15V and 18U). Unfortunately, *TRIM43* was undetectable, even in vehicle-treated 15V and 18U cells. The absence of *TRIM43*, a germline gene, is consistent with the notion that *DUX4* is required for *TRIM43* activation in FSHD muscles. Nevertheless, similar to our findings in FSHD patient myotubes, *miR-675-5p* was increased with each small molecule treatment in unaffected cells (Fig. 9 and Supplementary Fig. 19a). As an alternative approach to address this question, we over-expressed *miR-675-5p* in 15A FSHD myoblasts, and then treated differentiated cells with vehicle, β-estradiol, β-estradiol+MPA, or melatonin. The reasoning here was that in the presence of saturating *miR-675* levels, the drugs would be additive to further reduce *TRIM43* if they operated through a different mechanism. As expected, *miR-675-5p* levels significantly increased in *miR-675*-transfected cells (Supplementary Fig. 19b). Addition of the *miR-675*-upregulating drugs further increased *miR-675* levels. In each case, *DUX4* mRNA was significantly reduced compared to the untransfected, vehicle-treated control (Supplementary Fig. 19b). Although *TRIM43* levels were significantly decreased in all cells expressing increased levels, we measured no significant difference between *TRIM43* expression among *miR-675* treated cells, regardless of drug or vehicle treatment.

Supplementary Note 9. Potential effects of β-estradiol, β-estradiol+MPA, or melatonin on myoblast differentiation.

DUX4 levels typically increase upon differentiation of FSHD patient myoblasts into myotubes. In our experimental design, we differentiated myoblasts for 4-5 days prior to adding drugs, but nevertheless measured the impact of β-estradiol, β-estradiol+MPA, or melatonin on markers of muscle differentiation (*MYH2* and *MYOG*). None of the drug treatments significantly affected MYH2 or MYOG levels in 15A, 17A, or 18A myotubes (Supplementary Fig. 19c).

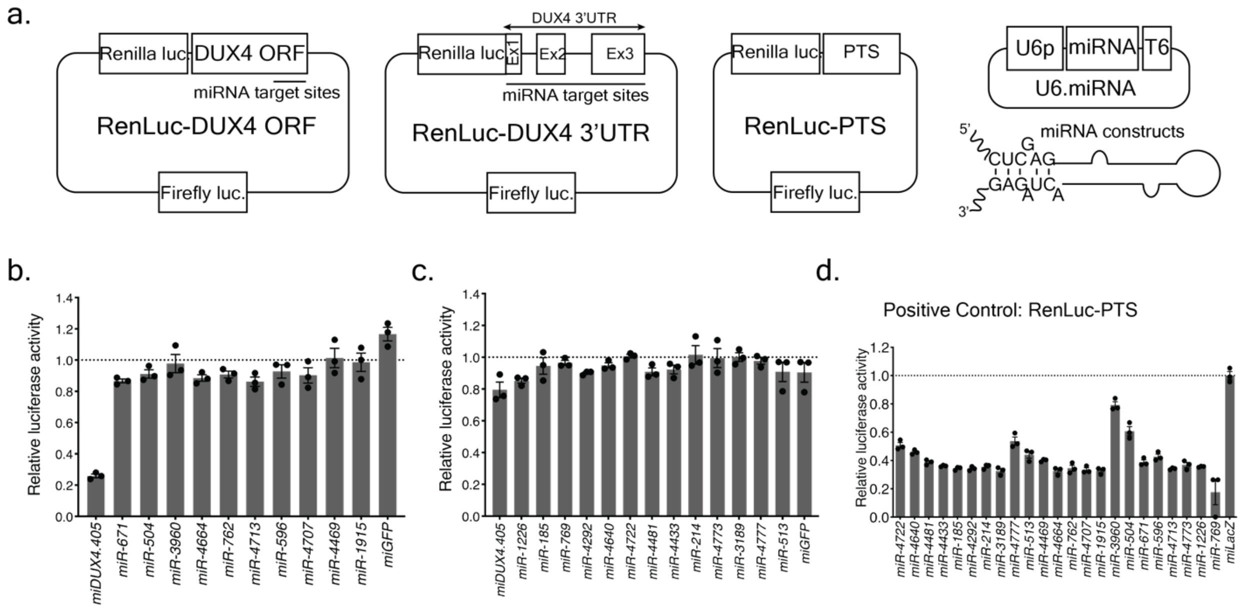

Supplementary Figure 1: Luciferase assays show miRbase-predicted miRNAs do not target *DUX4* sequences. a. Luciferase reporter plasmids. Three reporter plasmids encoding perfect target sites (PTS) for each miRNA (RenLuc-PTS), or the *DUX4 ORF* (RenLuc-DUX4 ORF), or *DUX4 3'UTR* (RenLuc-DUX4 3'UTR), cloned as a 3'UTR sequence of the *Renilla* luciferase gene. A separate firefly luciferase gene was included as a transfection control. We also show here the U6-driven expression plasmid and secondary structure scaffold of all tested miRNA constructs. Plasmids were co-tranfected into HEK293 cells and dual luciferase assays performed 24 hrs later. **b.** and **c.** The top 23 miRbase-predicted microRNAs were tested for their ability to silence *DUX4* expression using the dual-luciferase assay. A *Renilla*/*Firefly* activity ratio (Relative luciferase activity) below 1 indicates gene silencing of *DUX4* sequences. When normalized to the *miGFP* negative control, reduction of relative *Renilla* luciferase activity was less than 25% for 7 out of the 23 miRNAs targeting the *DUX4 ORF*, indicating a very poor inhibition efficiency (*mir-671*, **, P=0.0011; *mir-504*, **, P=0.0072; *mir-4664*, **, P=0.0024; *mir-762*, **, P=0.0061; *mir-4713*, ***, P=0.0009; *mir-596*, *, P=0.0139; *mir-4707*, **, P=0.0049). None of the miRbase microRNAs predicted to target the *DUX4 3'UTR* significantly silenced *DUX4* sequences

(P=0.9 for miRNAs). In contrast, the positive control *miDUX4.405* sequence reduced relative *Renilla* luciferase activity by 80% (P<0.0001) (3, 6). Results were reported as the average relative *Renilla* luciferase activity ± SEM (N=3 independent experiments). **d.** To confirm functionality of each microRNA, we tested their effectiveness to reduce perfect target sites, using the RenLuc-PTS construct. Readings were normalized to the miLacZ negative control. All miRNAs were functional as they reduced the relative *Renilla* luciferase activity by 20 to 60% (P<0.0001). Results were reported as the average relative *Renilla* luciferase activity ± SEM (N=3 independent experiments). One-way ANOVA followed by Tukey's (**b.** and **c.**) or Dunnett's (**d.**) multiple comparison tests were performed for statistical analyses. Source data are provided as a Source Data file.

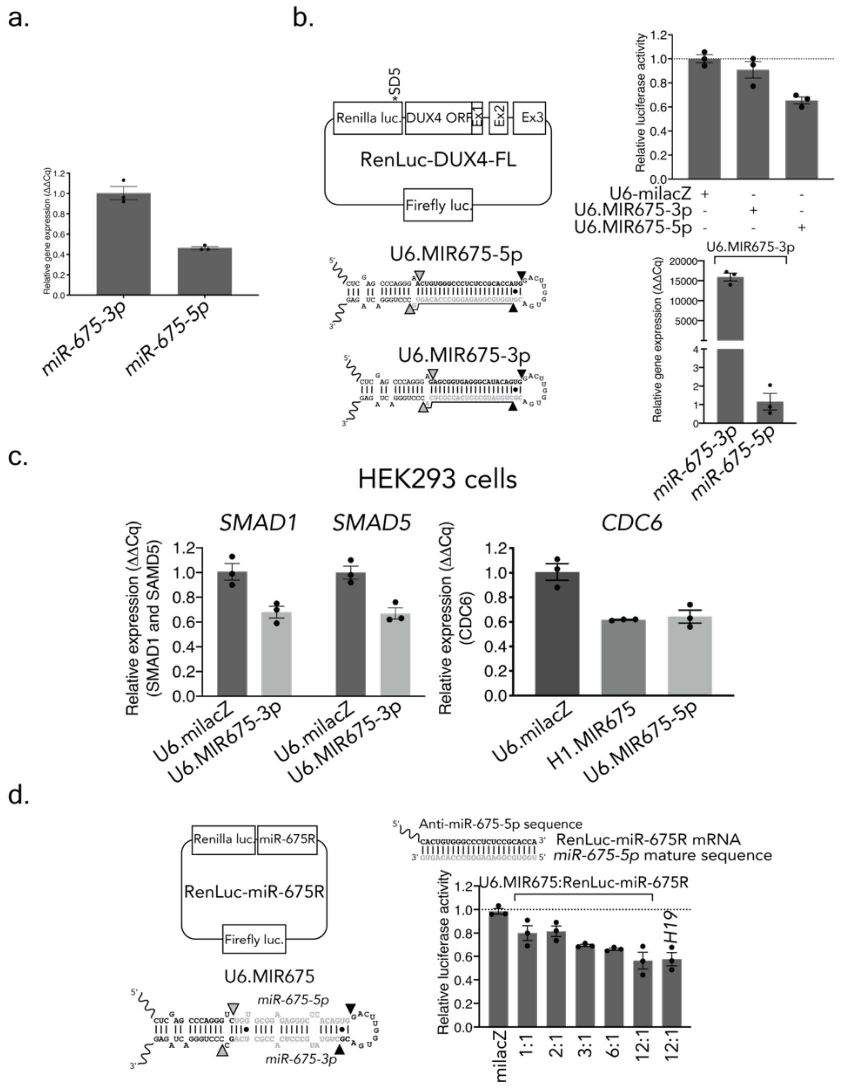

Supplementary Figure 2: *miR-675-5p*, but not *miR-675-3p*, targets *DUX4*. a. QPCR analysis of *miR-675* expression in HEK293 cells transfected with *hsa-H19* lncRNA (CMV.H19). Both *miR-675-3p* and *miR-675-5p* were processed as mature miRNAs from the primary *H19*/*miR-675* transcript, with mature *miR-675-3p* being 2.20±0.15-fold more abundant than *miR-675-5p* in this assay (P=0.0012). Gene expression was normalized to the house keeping gene *RPL13A*. Results were reported as the average relative gene expression ± SEM (N=3 independent experiment) normalized to *miR-675-3p*. Unpaired two-tailed t test was performed for statistical analyses. **b.** Dual-luciferase assay using constructs designed to specifically produce U6.MIR675-3p or

U6.MIR675-5p. A 40:1 molar ratio of miRNA to luciferase plasmid was used. *MiR-675-5p* reduced *Renilla* luciferase activity from the DUX4-FL construct by 35±4% (P=0.0038), while the *miR-675-3p* sequences had no significant impact even though U6.MIR675-3p expressed high levels of *miR-675-3p* relative to the negative control *miR-675-5p* levels (QPCR graph at bottom right (P<0.0001)). All readings were normalized to the miLacZ negative control. Results were reported as the average relative *Renilla* luciferase activity ± SEM (N=3 independent experiments). One-way ANOVA followed by Dunnett's multiple comparison tests were performed for statistical analyses. **c.** Both strands of *miR-675* are functional to silence known targets. QPCR confirmed that *miR-675*-3p targets *SMAD1* and *SMAD5* and *miR-675-5p* silenced *CDC6* in transfected HEK293 cells. Specifically, U6.MIR675-3p significantly reduced *SMAD1* (32±6%; P=0.0052) and *SMAD5* levels (33±6%; P=0.0049), while H1.MIR675 and U6.MIR675-5p repressed *CDC6* levels by an average of 39±4% (P=0.0025) and 36±7% (P=0.0036), respectively. Results were normalized to the housekeeping gene *RPL13A*, and reported as relative gene expression (ΔΔCq) ± SEM (N=3 independent experiments) relative to gene expression in cells transfected with U6.miLacZ, with each QPCR assay performed in triplicate. For *SMAD1* and *SMAD5* expression, two-way ANOVA followed by Sidak's multiple comparison tests were performed for statistical analyses. For *CDC6* expression, one-way ANOVA followed by Dunnett's multiple comparison tests were performed for statistical analyses. **d.** Dual-luciferase assay using a perfect *miR-675-5p* binding site as target sequence (*miR-675R*). U6.MIR675 reduced the relative *Renilla* luciferase activity in a dose-dependent manner reaching a maximum inhibition of 43±7% (P<0.0001), and CMV.H19 reduced the relative *Renilla* luciferase activity by 42±6% (P=0.0001). All readings were normalized to the miLacZ negative control. Results were reported as the average relative *Renilla* luciferase activity ± SEM (N=3 independent experiments). One-way ANOVA followed by Dunnett's multiple comparison tests were performed for statistical analyses. Source data are provided as a Source Data file.

a.

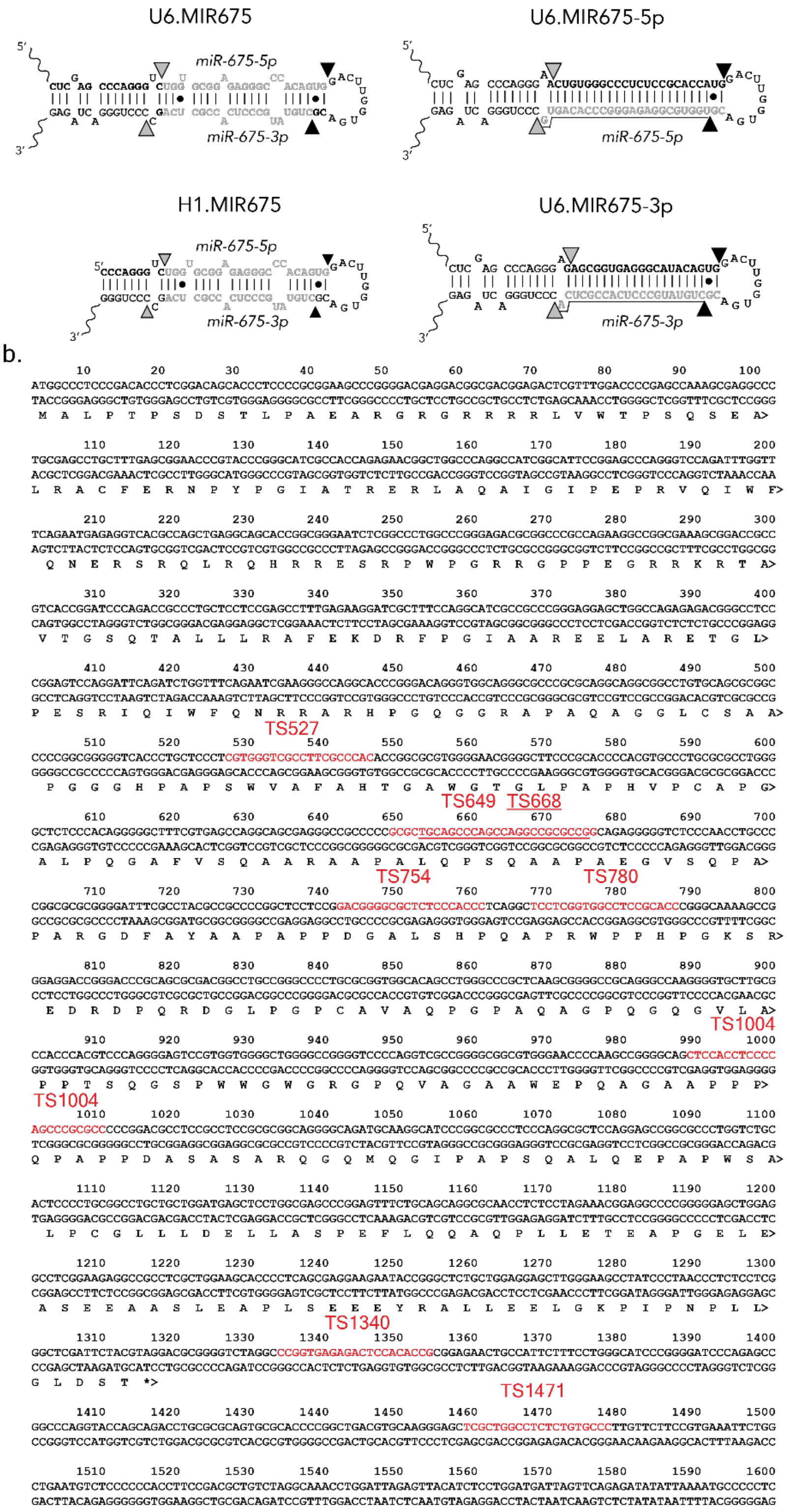
U6.MIR675
U6.MIR675-5p
H1.MIR675
U6.MIR675-3p
miR-675-5p
miR-675-3p
5′
3′
b.
TS527
TS649
TS668
TS754
TS780
TS1004
TS1340
TS1471

Supplementary Figure 3: *miR-675* binding sites in *DUX4* sequence. a. Stem loop structures of constructs designed to express both strands of *miR-675*, or individual strands *miR-675-5p* and *miR-675-3p*. **b.** DUX4 sequence (*DUX4 ORF+3'UTR* without introns). The validated *miR-675-5p* binding sites are highlighted in red. TS649 and TS668 overlap, with TS668 underlined.

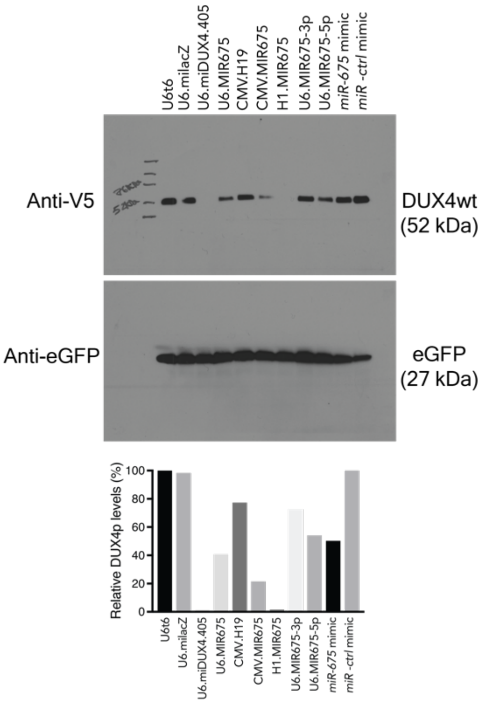

Supplementary Figure 4: Raw western blot replicate with quantification.

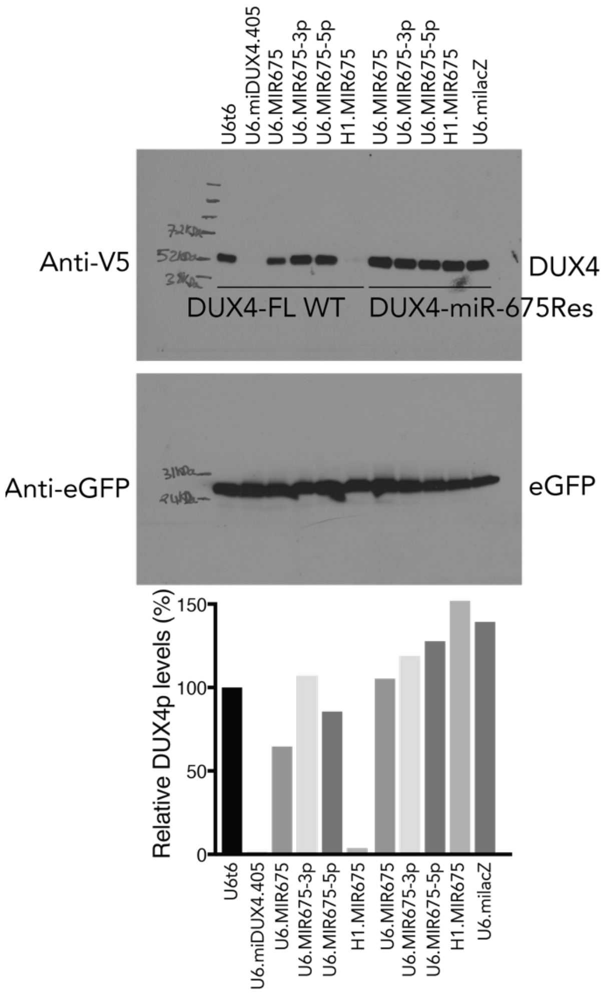

Supplementary Figure 5: Raw western blot replicate with quantification.

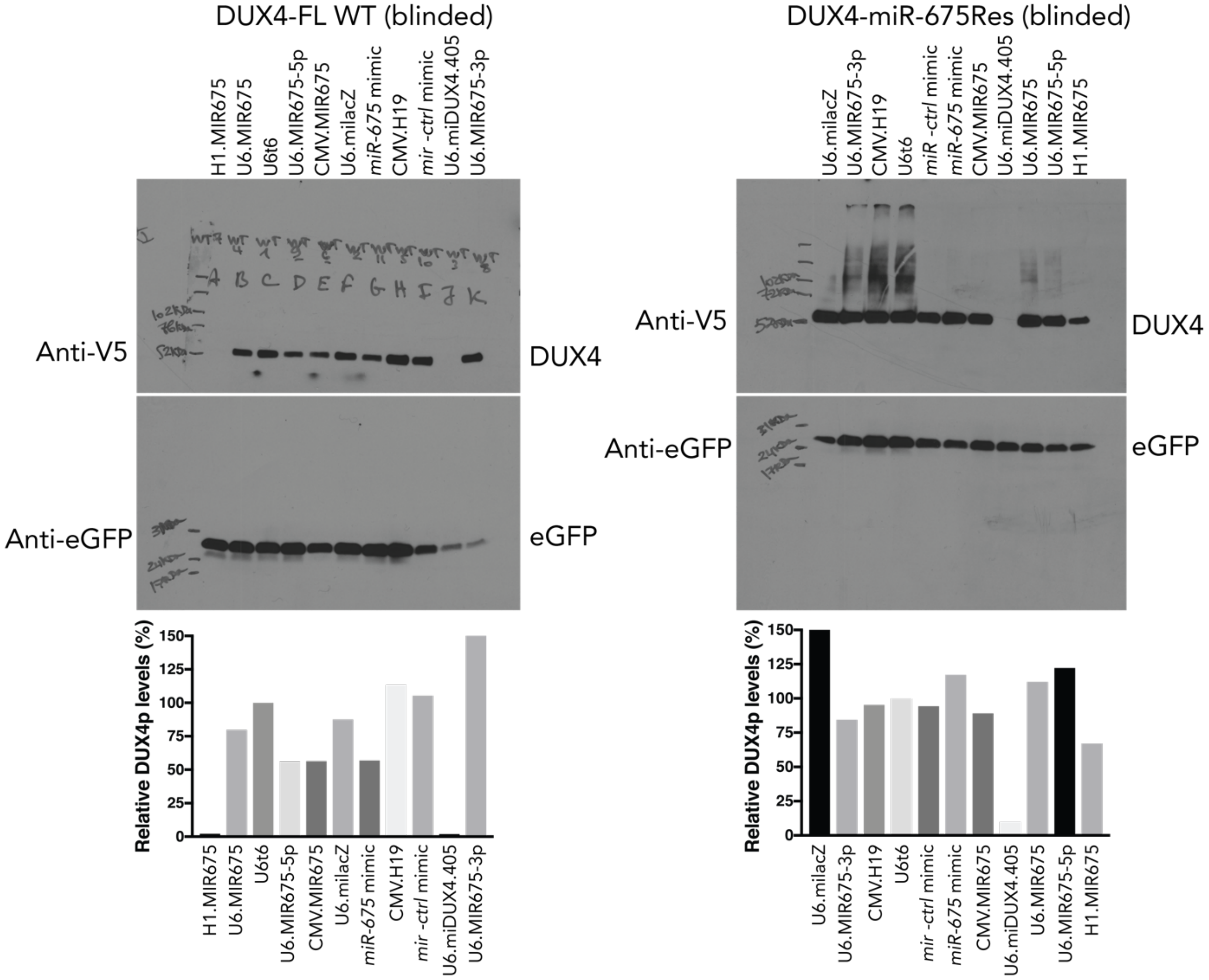

Supplementary Figure 6: Raw western blot replicate with quantification.

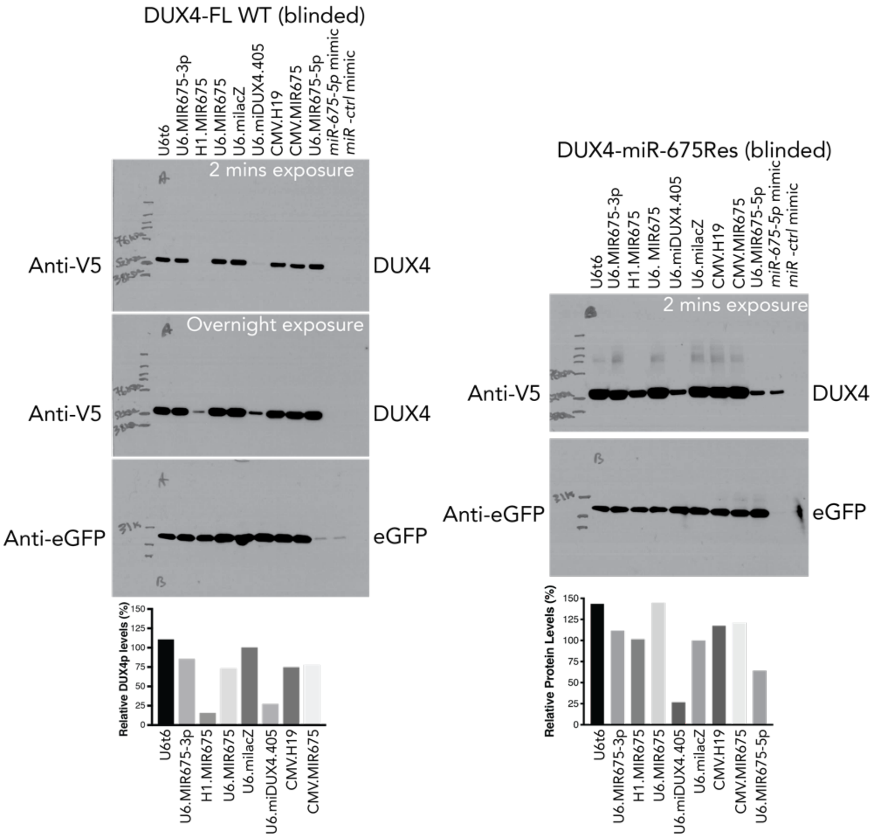

Supplementary Figure 7: Raw western blot replicate with quantification.

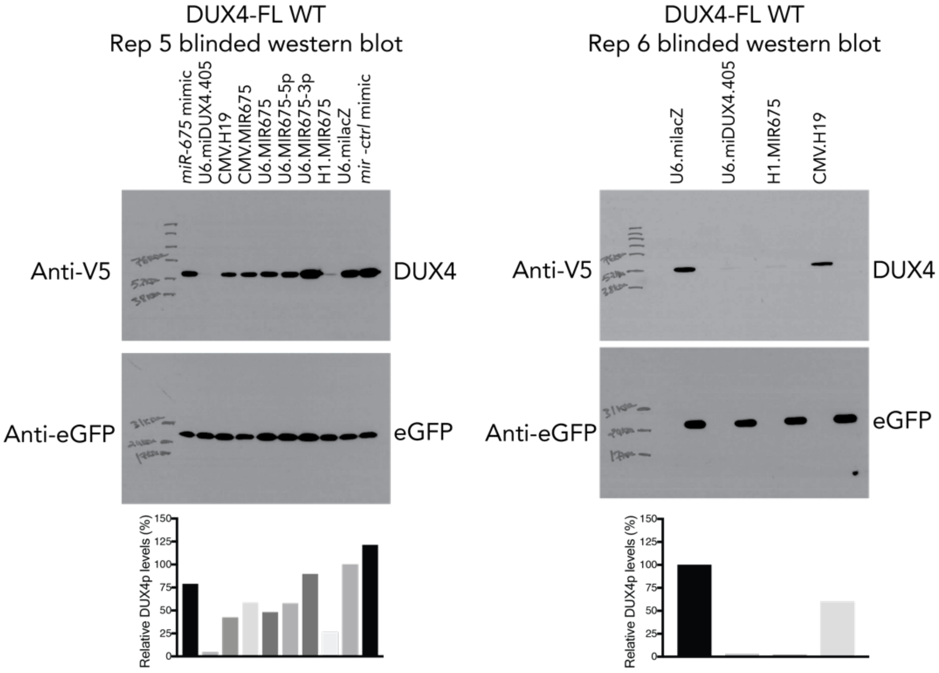

Supplementary Figure 8: Raw western blot replicate with quantification.

Supplementary Figures 4-8: U6.MIR675, H1.MIR675, U6.MIR675-5p and CMV.H19 reduce DUX4 protein level. Independent experiments showing 2 unblinded and 6 blinded western blots performed on protein extracts from HEK293 cells co-transfected with various constructs expressing *miR-675* and the indicated full-length V5-tagged wild-type or *miR-675*-resistant *DUX4* constructs. DUX4 expression constructs co-express eGFP from the same plasmid backbone, thereby allowing use of eGFP as a transfection control and reference gene for quantification. DUX4 protein levels were quantified relative to U6t6 or miLacZ samples in each experiment.

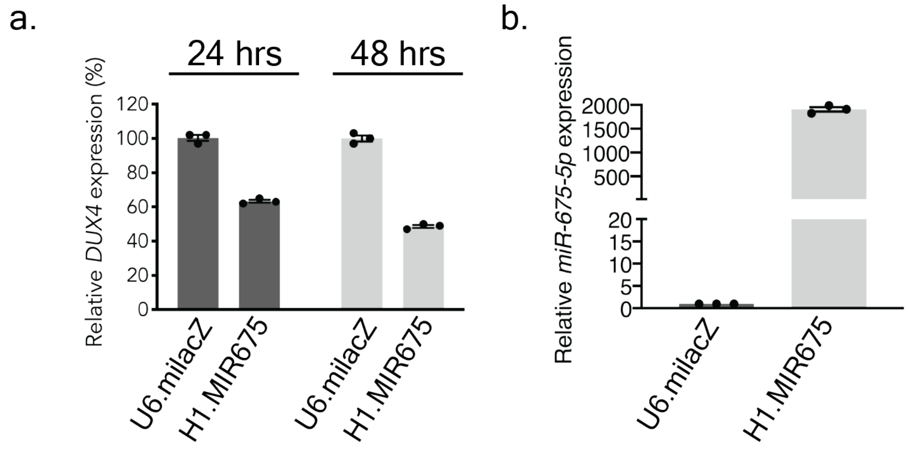

Supplementary Figure 9: H1.MIR675 reduces *DUX4* mRNA levels in co-transfected HEK293 cells. a. QPCR from RNAs harvested from HEK293s transfected with H1.MIR675 or control miLacZ in a 3 to 1 molar ratio to *DUX4* expression plasmid. *DUX4* values were normalized to eGFP (which is present as transfection control on the DUX4 expression plasmid). *DUX4* levels were reduced in *miR-675*-treated cells by 37±2% and 51±2% at 24h and 48h, respectively ($P<0.0001$). Results were reported as the average relative DUX4 expression ± SEM (N=3 independent experiments). Two-way ANOVA followed by Sidak's multiple comparison tests were performed for statistical analyses. **b.** QPCR showed ~2000-fold increase in *miR-675-5p* following

over-expression in HEK293s (P<0.0001, two-tailed unpaired t test). Results were reported as the average relative *miR-675-5p* expression (ΔΔCq) ± SEM (N=3 independent experiments). Source data are provided as a Source Data file.

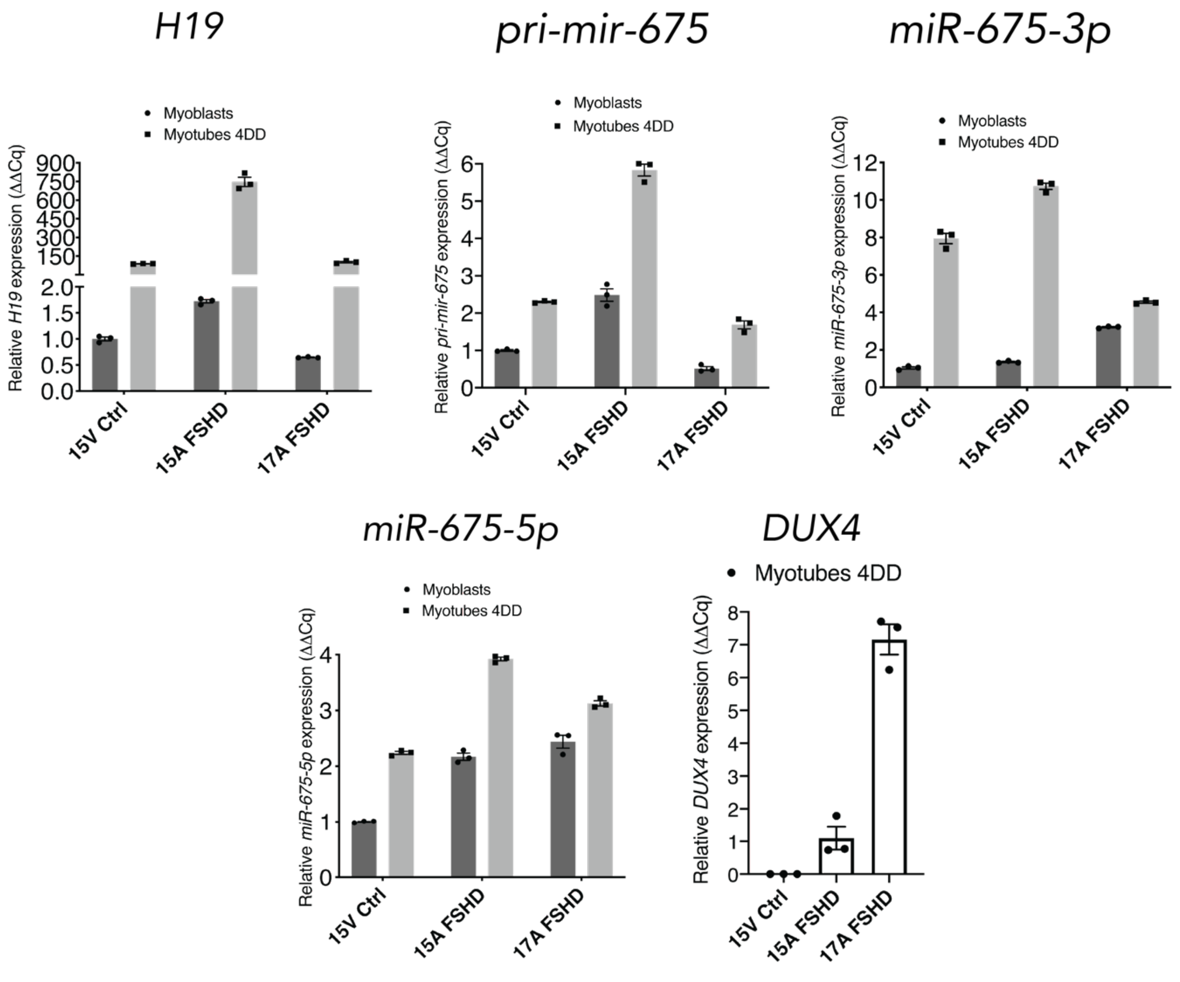

Supplementary Figure 10: *DUX4*, *miR-675* and its precursors *H19* and *pri-mir-675* were differentially expressed in human skeletal muscle myotubes. QPCR measurement of indicated transcripts in 15V, 15A and 17A myoblasts and myotubes (differentiated 4 days). *H19*, *pri-mir-675*, *miR-675-3p* and *miR-675-5p* levels significantly increased upon differentiation in all cell lines, as follows: All values were significant with P<0.0001. In 15V and 17A cells, *H19* had a P=0.0048 and P=0.0016, respectively. Two-way ANOVA followed by Sidak's multiple comparison tests were performed for statistical analyses. In addition, *DUX4* gene expression was measured

in differentiated 15V, 15A, and 17A myotubes. *DUX4* expression was 6.5±2.1-fold (P<0.0001) higher in 17A myotubes compared to 15A myotubes, and not detected in unaffected 15V myotubes. One-way ANOVA followed by Tukey's multiple comparison tests were performed for statistical analyses. Results were reported as gene expression (ΔΔCq) ± SEM (N=3 independent experiments) relative to 15A myotubes for DUX4, with each QPCR assay performed in triplicate. All results were normalized to the *RPL13A* reference gene. Source data are provided as a Source Data file.

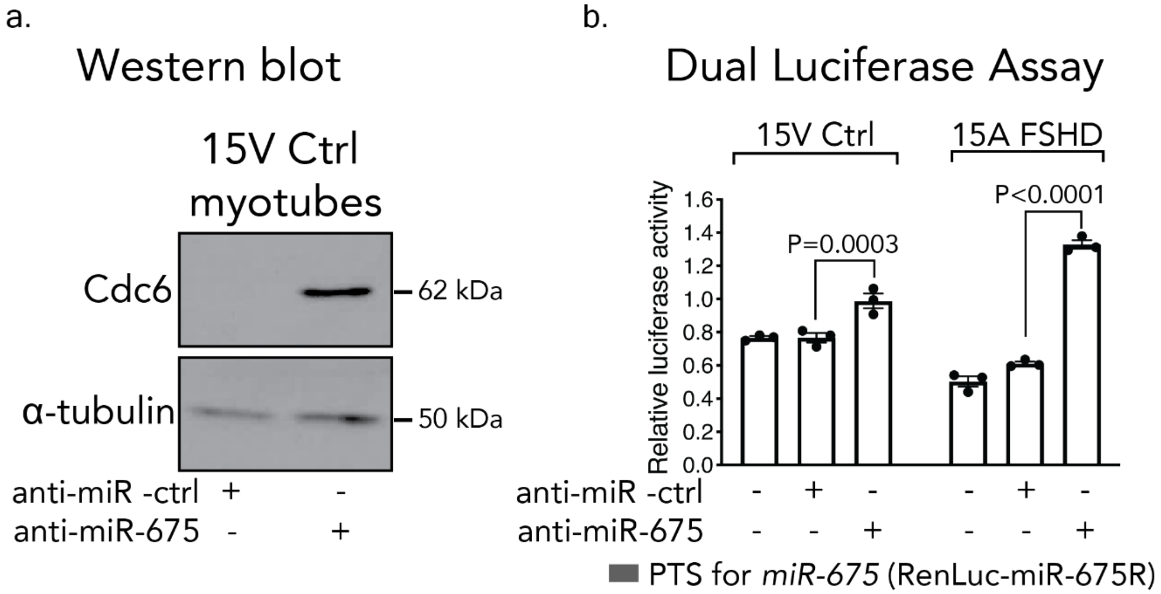

Supplementary Figure 11: Validation of *miR-675* antagomirs *in vitro*. The anti-miR-675 antagomir was used at a final concentration of 300 nM. a. Western blot to detect Cdc6 protein, a known *miR-675-5p* target, or the loading control α-tubulin, in 4-day differentiated 15V unaffected myotubes. Cdc6 was only detected in myotubes transfected with *anti-miR-675* (Supplementary Fig. 12 for uncropped gel). b. Dual-luciferase assay using a reporter containing Renilla luciferase with a perfect target site (PTS) for *miR-675-5p* (RenLuc-miR-675R). Luciferase activity was measured 48 hours after co-transfection of 15A or 15V myoblasts with the reporter plasmid and 300 nM of anti-miR-675 or anti-miR -ctrl. Anti-miR-675 caused significant de-repression of *Renilla*

luciferase in both cell lines, indicating that endogenous *miR-675-5p* functioned to inhibit the reporter. In 15V and 15A, the relative *Renilla* luciferase activity increased by 1.3±0.1-fold (P=0.0003) and 2.2±0.1-fold (P<0.0001), respectively. Two-way ANOVA followed by Tukey's multiple comparison tests were performed for statistical analyses. All data represent mean normalized *Renilla* luciferase activity ± SEM (N = 3 independent experiments). Source data are provided as a Source Data file.

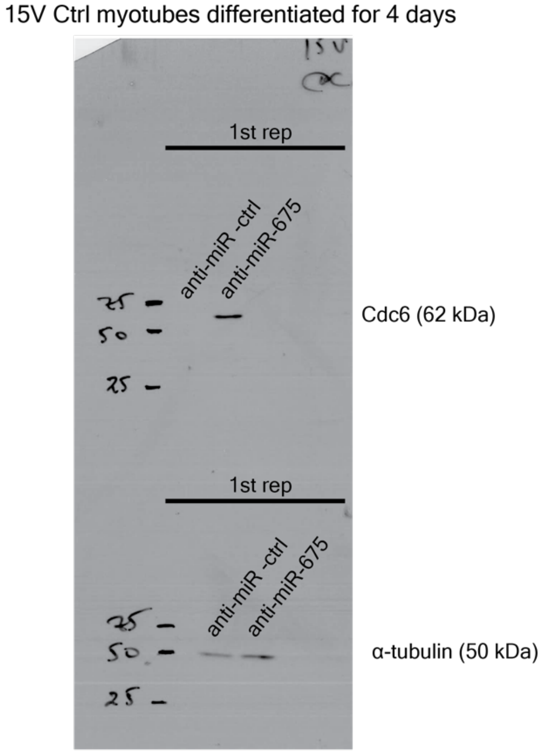

Supplementary Figure 12: Uncropped western blot image to detect Cdc6 and alpha-tubulin proteins in 15V Ctrl myotubes transfected with indicated anti-miRs.

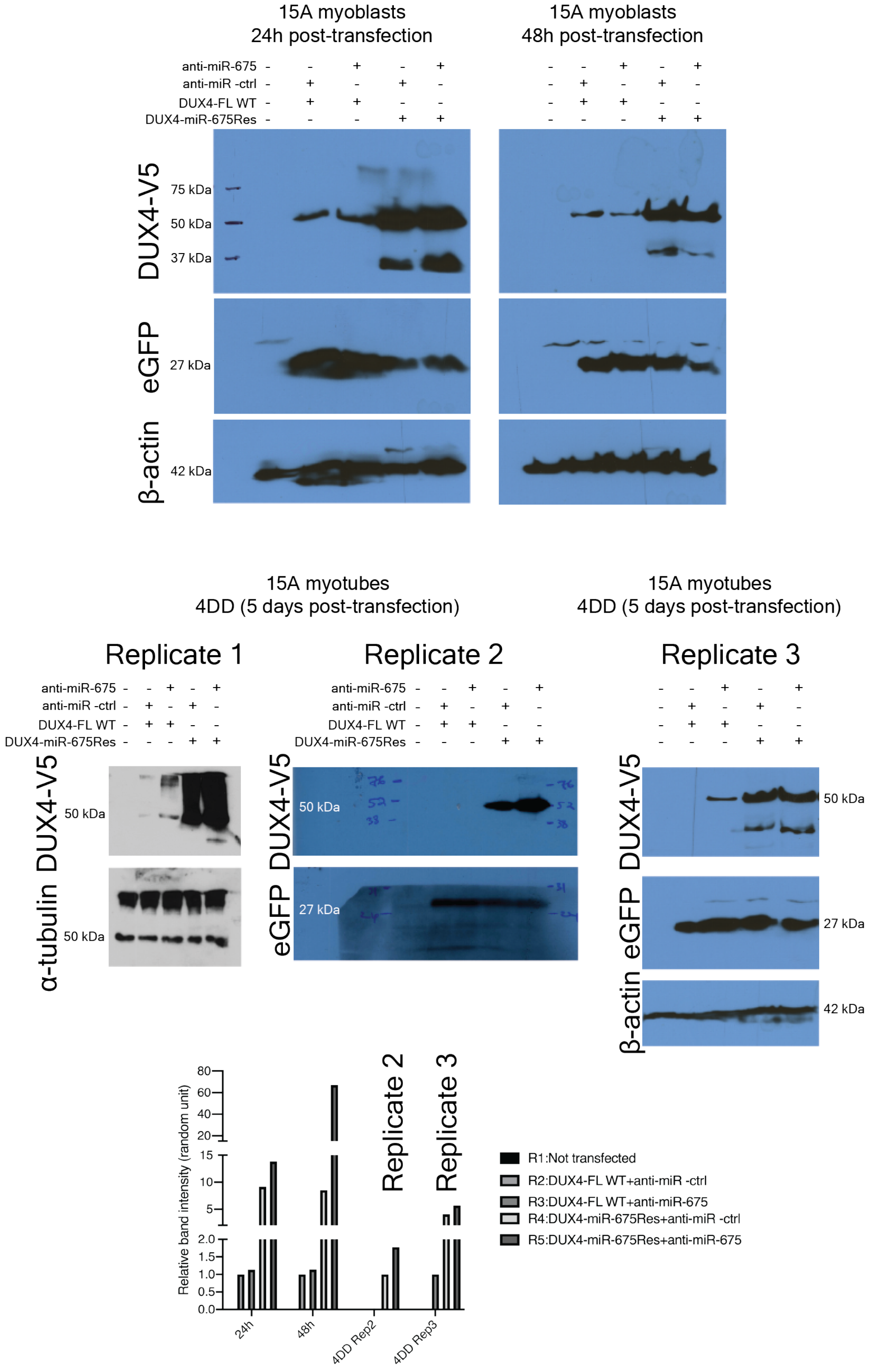

Supplementary Figure 13: Supplement to Figure 5c western blot. Three repeated western blots performed on protein extracts from 15A FSHD myoblasts and myotubes co-transfected with

anti-miR-675-5p*, DUX4-FL (WT)* and *DUX4-miR-675Res* constructs. Myoblasts were collected 24 and 48 hours after transfection. Myotubes were collected 4 days after differentiation (5 days after transfection). For Rep. 1 and 2, alpha-tubulin was used as a reference gene, and was detected using the alpha-tubulin rabbit polyclonal antibody (1:500 in 5% milk TBST buffer, ab15246; Abcam). DUX4 protein was detected using an anti-V5 antibody (HRP-coupled mouse monoclonal antibody used at 1:5,000 in 5% milk TBST buffer). For Rep. 3 the 15A myoblasts were transfected with CMV.DUX4-FL/CMV.eGFP or CMV.DUX4-miR-675Res plasmids, both of which co-express eGFP from the same plasmids. In this experiment, eGFP was used as transfection control and a reference gene for quantification. In this replicated western blot, DUX4 protein was also detected using an anti-V5 antibody. β-actin was used as an endogenously expressed protein reference. β-actin was detected using an anti-mouse monoclonal antibody (1:1000 in 5% milk TBST buffer, SIGMA). The graph shows quantification of DUX4 protein levels in all tested conditions. Source data are provided as a Source Data file.

a.

H&E staining in C57BL/6 TA

7098

7098-TA/L: H1.miR-675 (5E+10 particles)-4X

7098-TA/R: Saline-4X

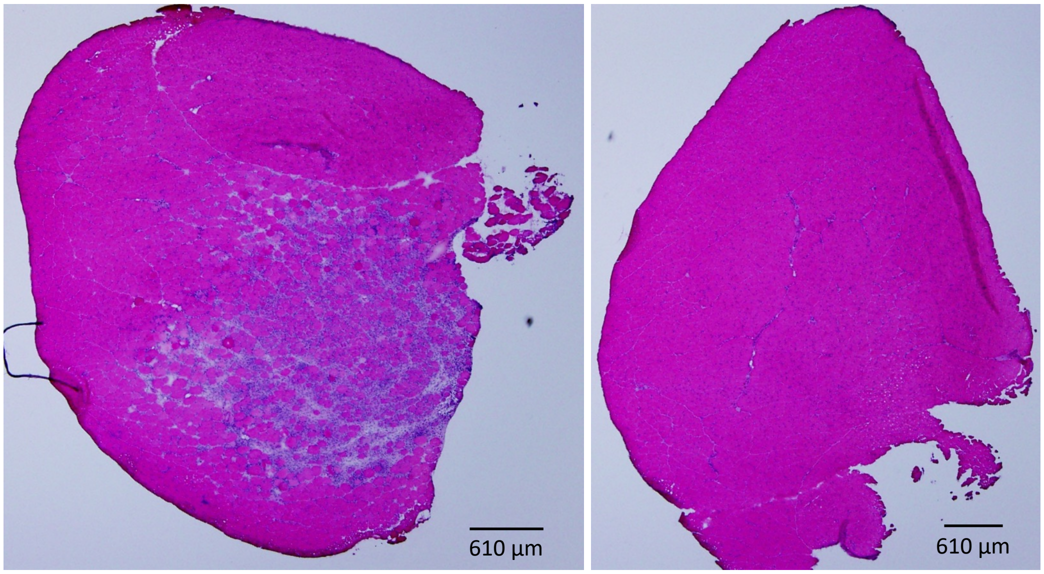

b.

H&E staining in C57BL/6 TA

7098

7098-TA/L: H1.miR-675 (5E+10 particles)-10X

7098-TA/R: Saline-10X

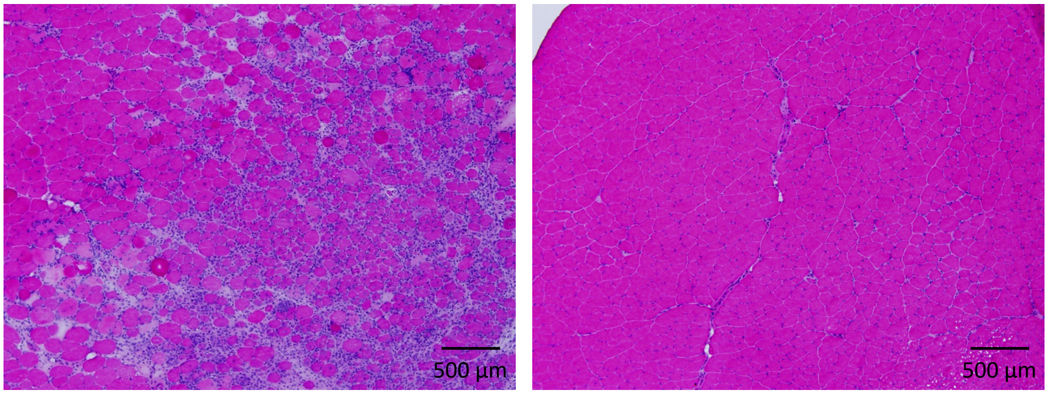

c.

7098

H&E staining in C57BL/6 TA
7098-TA/L: H1.miR-675 (5E+10 particles)-20X

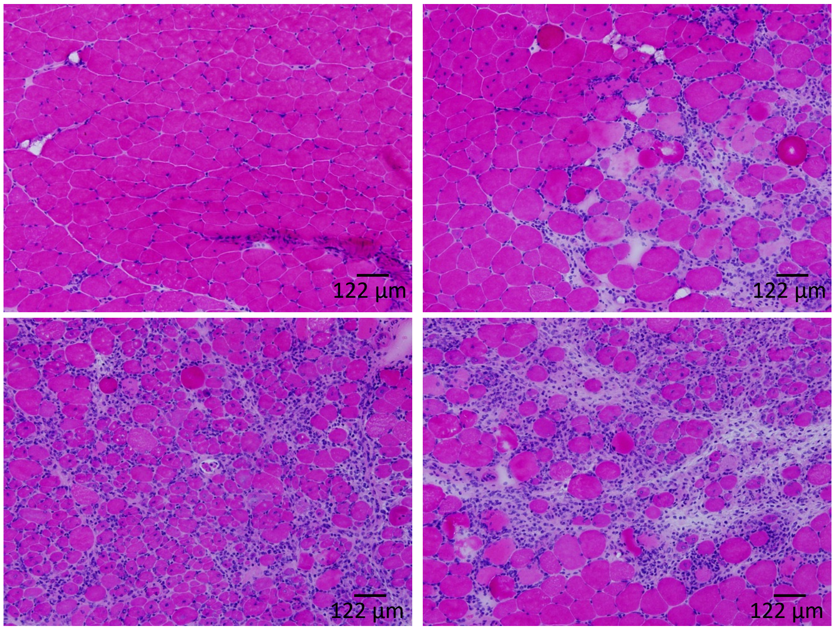

d.

7098

H&E staining in C57BL/6 TA
7098-TA/R: Saline-20X

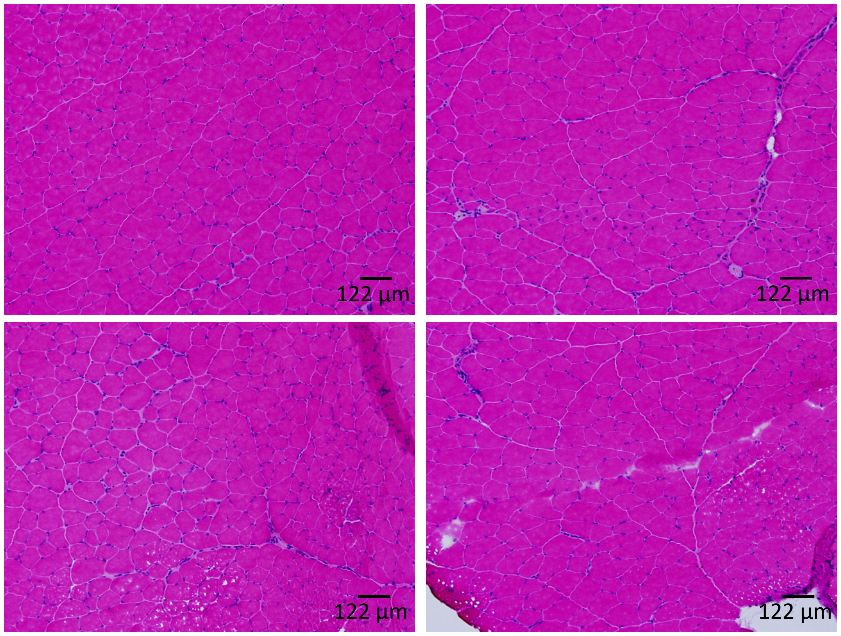

e. H&E staining in C57BL/6 TA

7099

7099-TA/L: H1.miR-675 (5E+10 particles)-4X   7099-TA/R: Saline-4X

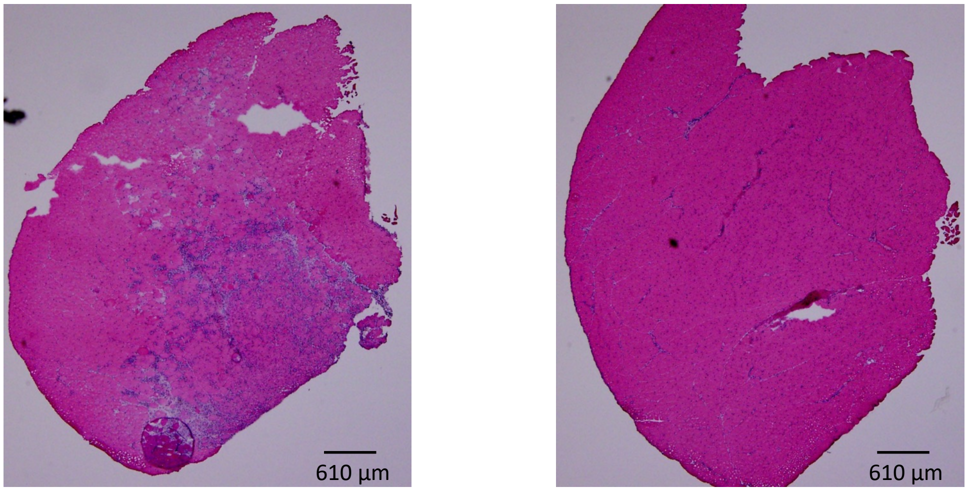

f. H&E staining in C57BL/6 TA

7099

7099-TA/L: H1.miR-675 (5E+10 particles)-10X   7099-TA/R: Saline-10X

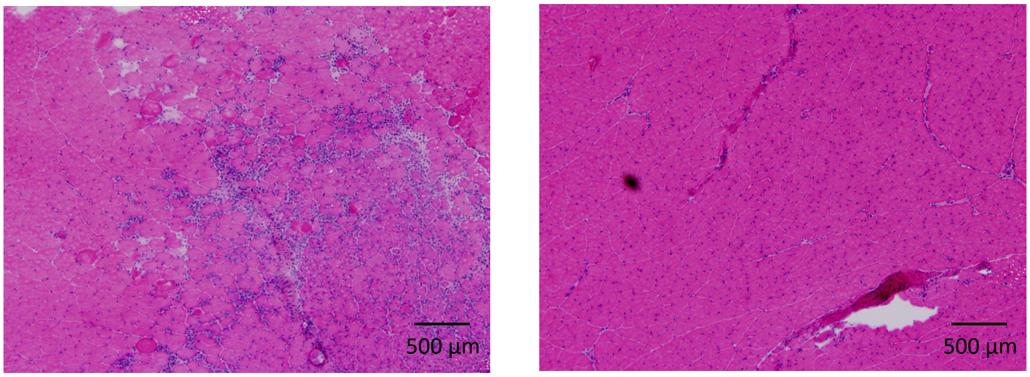

g.

7099

H&E staining in C57BL/6 TA

7099-TA/L: H1.miR-675 (5E+10 particles)-20X

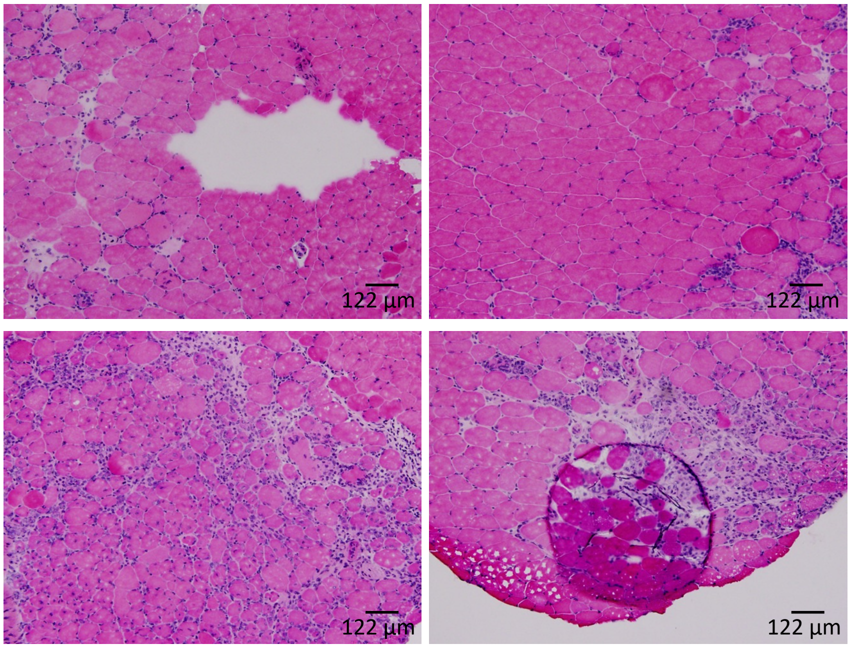

h.

7099

H&E staining in C57BL/6 TA

7099-TA/R: Saline-20X

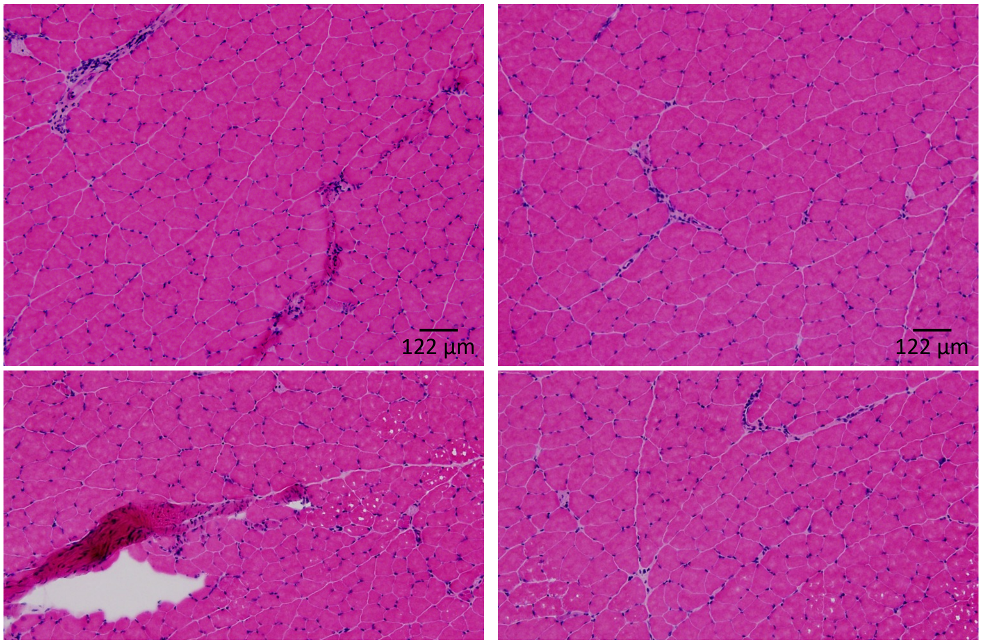

i.

H&E staining in C57BL/6 TA

7100

7100-TA/L: H1.miR-675 (5E+10 particles)-4X

7100-TA/R: Saline-4X

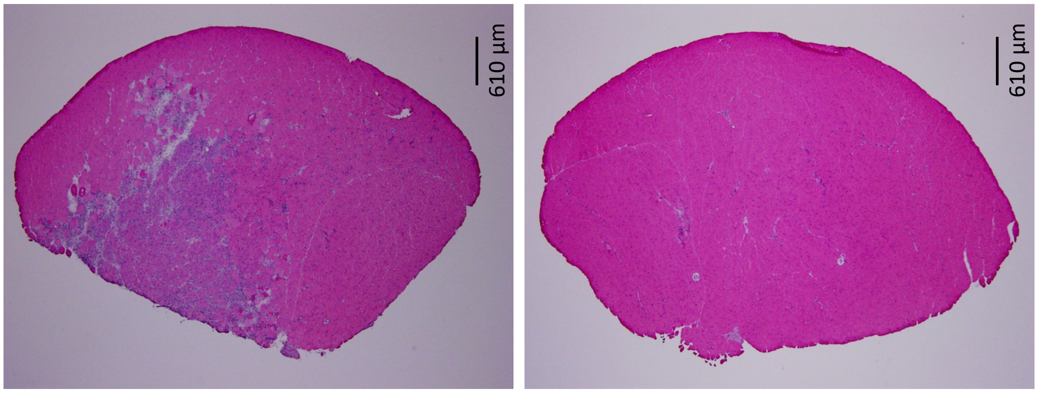

j.

H&E staining in C57BL/6 TA

7100

7100-TA/L: H1.miR-675 (5E+10 particles)-10X

7100-TA/R: Saline-10X

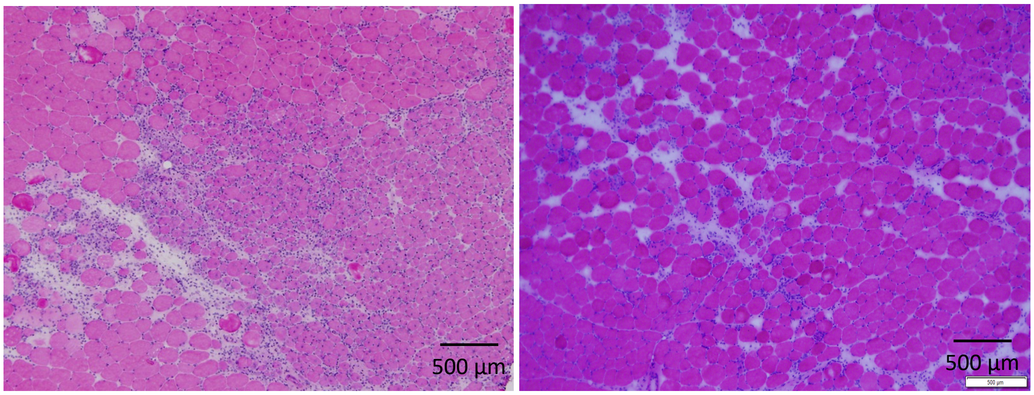

k.

7100

H&E staining in C57BL/6 TA

7100-TA/L: H1.miR-675 (5E+10 particles)-20X

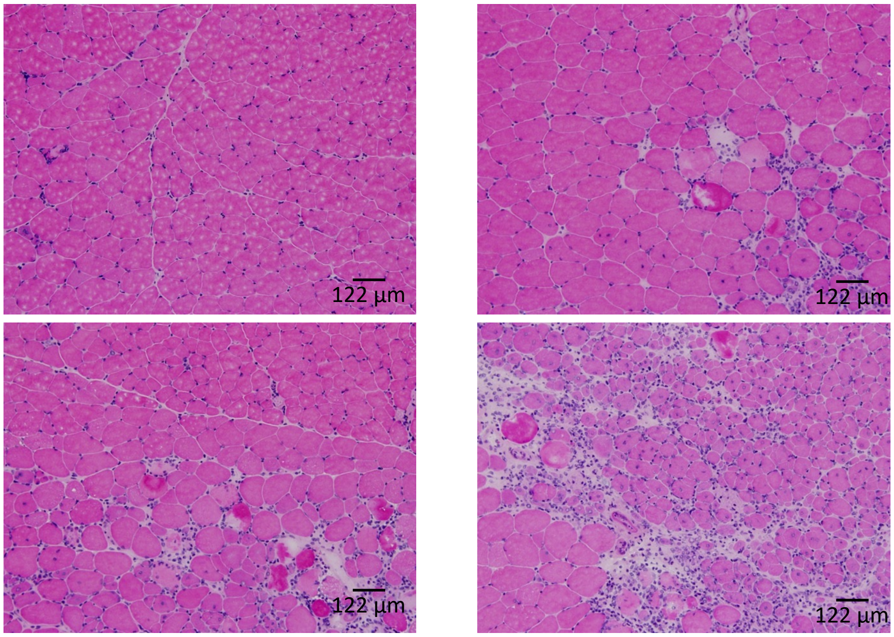

l.

7100

H&E staining in C57BL/6 TA

7100-TA/R: Saline-20X

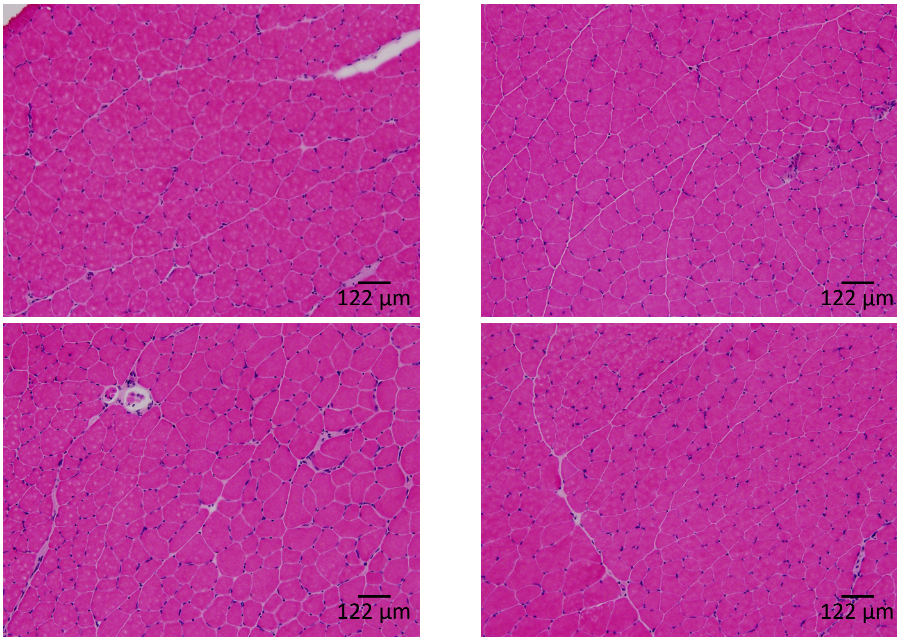

Supplementary Figure 14: a-l. Hematoxylin and eosin (H&E) staining of Tibialis anterior (TA) injected with scAAV.H1.MIR675. H&E staining of 10 µm muscle sections from C57BL/6 TA

mouse muscles injected with scAAV6.MIR675 construct expressing H1.MIR675 ($5x10^{10}$ particles) show toxicity (N=3).

a.

H&E staining: 2Wks in C57BL/6 TA
7162-TA/L: U6.miR-675-2 (5E+10 particles)+DUX4-FL (3E+09 particles)

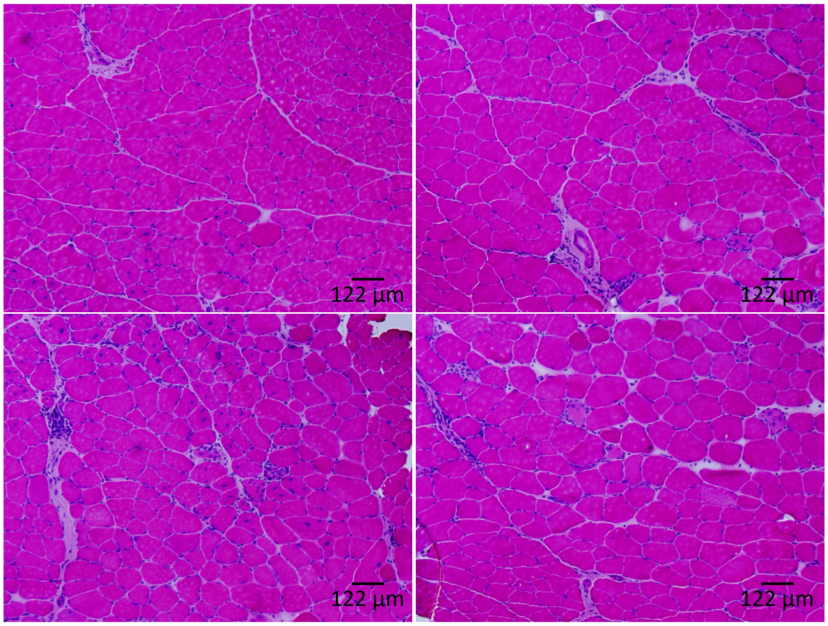

b.

H&E staining: 2Wks in C57BL/6 TA
7162-TA/R: U6.milacZ (5E+10 particles)+DUX4-FL (3E+09 particles)

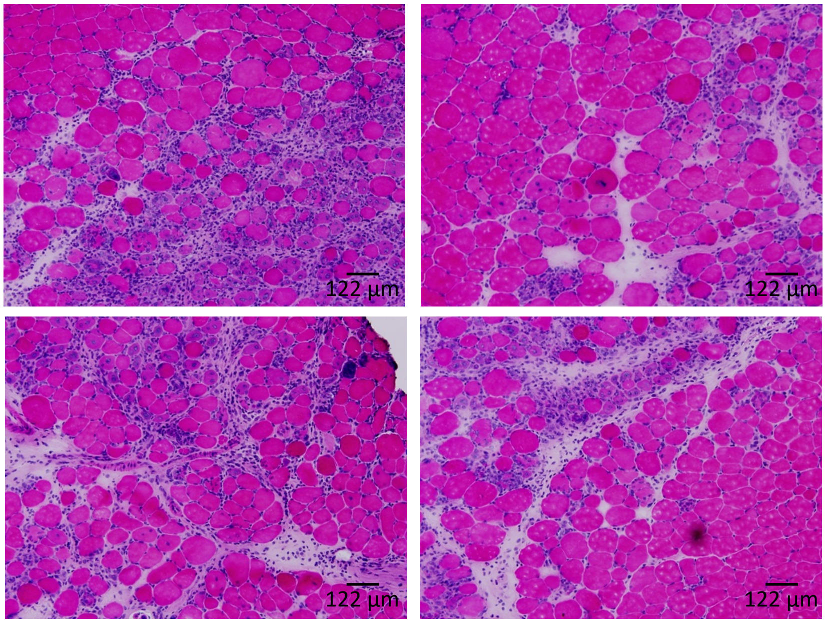

c.

H&E staining: 2Wks in C57BL/6 TA
7163-TA/L: U6.miR-675-2 (5E+10 particles)+DUX4-FL (3E+09 particles)

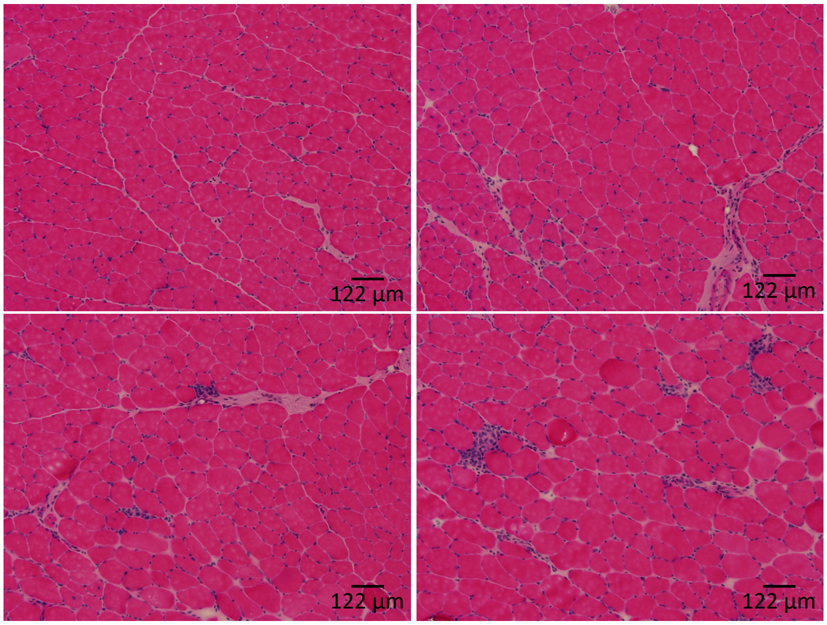

d.

H&E staining: 2Wks in C57BL/6 TA
7163-TA/R: U6.milacZ (5E+10 particles)+DUX4-FL (3E+09 particles)

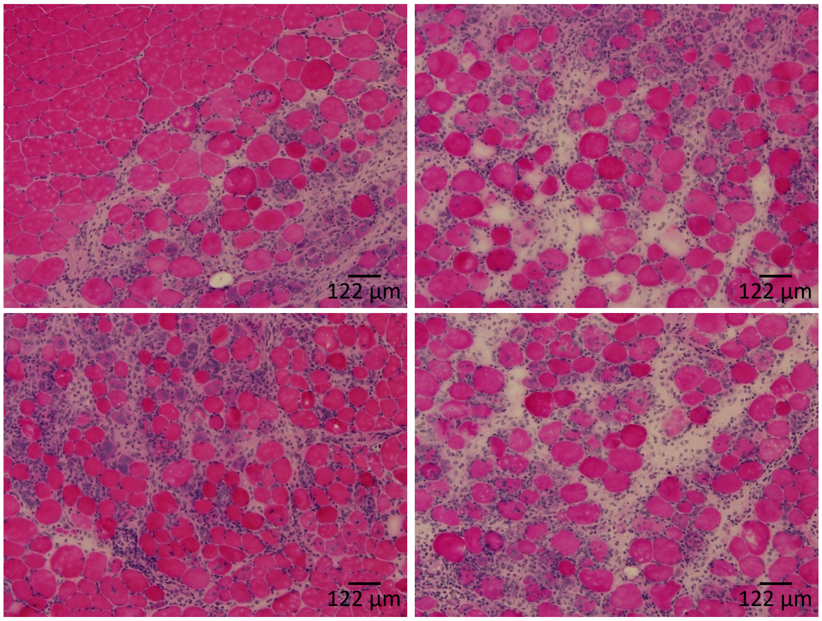

e.

H&E staining: 2Wks in C57BL/6 TA
7164-TA/L: U6.miR-675-2 (5E+10 particles)+DUX4-FL (3E+09 particles)

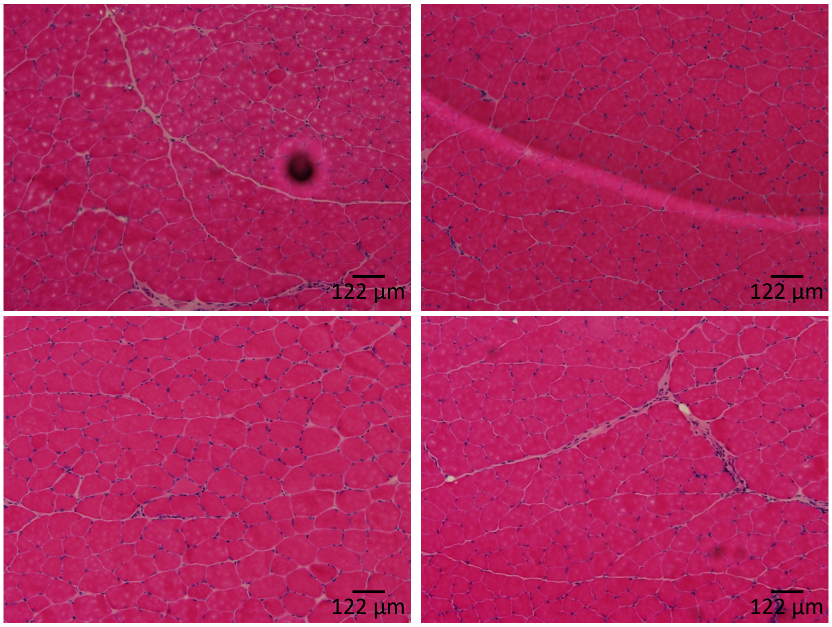

f.

H&E staining: 2Wks in C57BL/6 TA
7164-TA/R: U6.milacZ (5E+10 particles)+DUX4-FL (3E+09 particles)

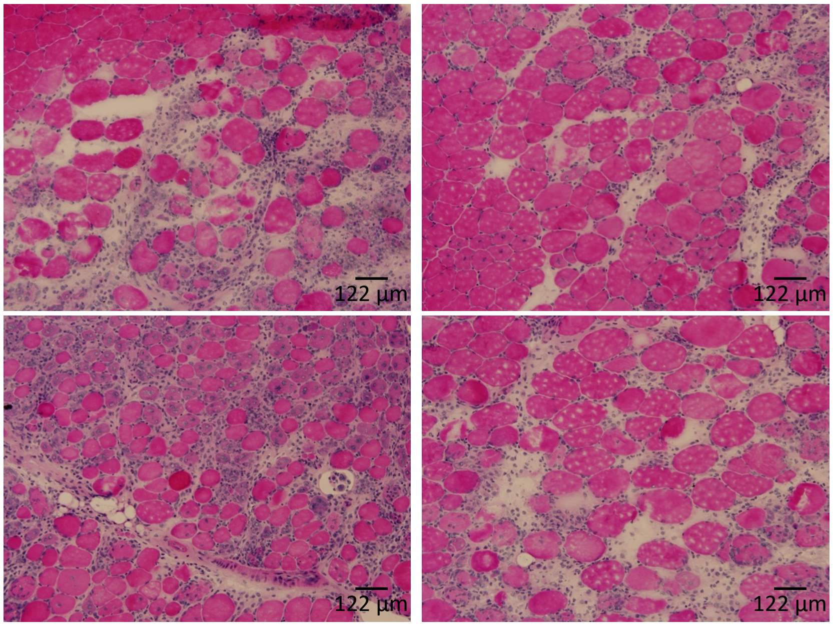

g.

H&E staining: 2Wks in C57BL/6 TA
7165-TA/L: U6.miR-675-2 (5E+10 particles)+DUX4-FL (3E+09 particles)

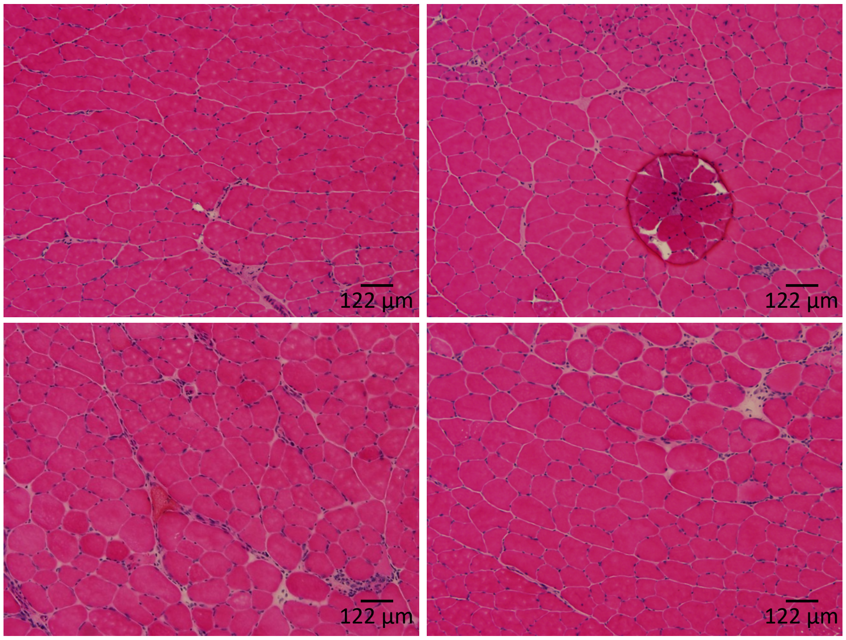

h.

H&E staining: 2Wks in C57BL/6 TA
7165-TA/R: U6.milacZ (3E+09 particles)+miR-675-2 (5E+10 particles)

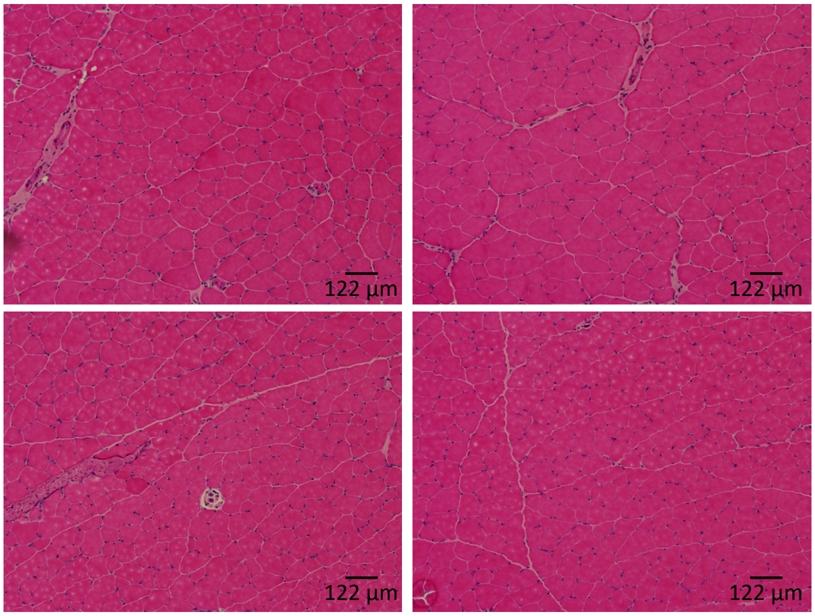

i.

H&E staining: 2Wks in C57BL/6 TA
7166-TA/L: U6.miR-675-2 (5E+10 particles)+DUX4-FL (3E+09 particles)

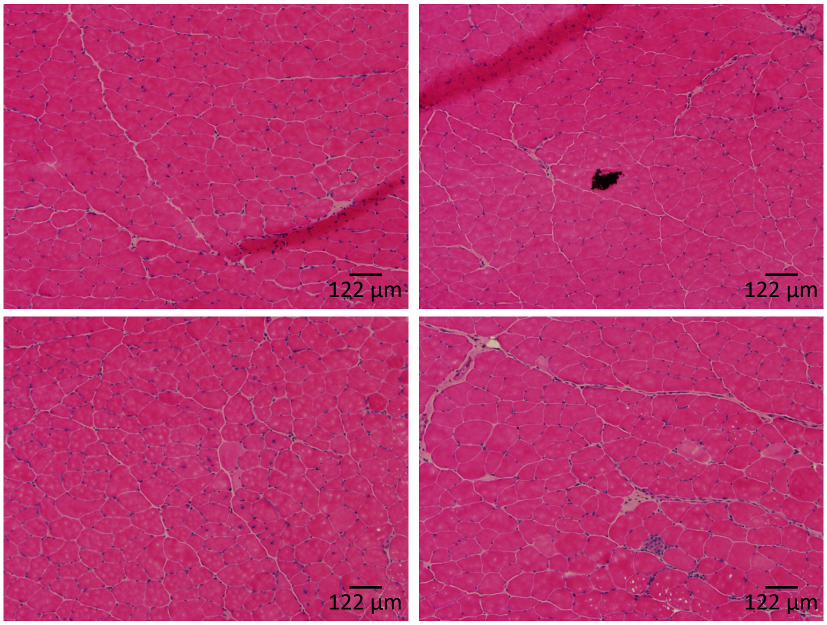

j.

H&E staining: 2Wks in C57BL/6 TA
7166-TA/R: U6.milacZ (3E+09 particles)+U6.miR-675-2 (5E+10 particles)

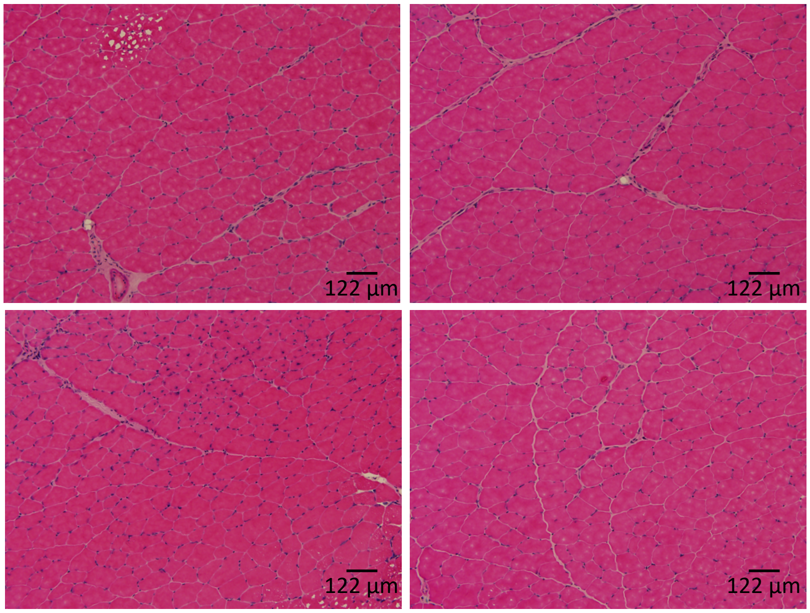

k.

H&E staining: 2Wks in C57BL/6 TA
7167-TA/L: U6.miR-675-2 (5E+10 particles)+DUX4-FL (3E+09 particles)

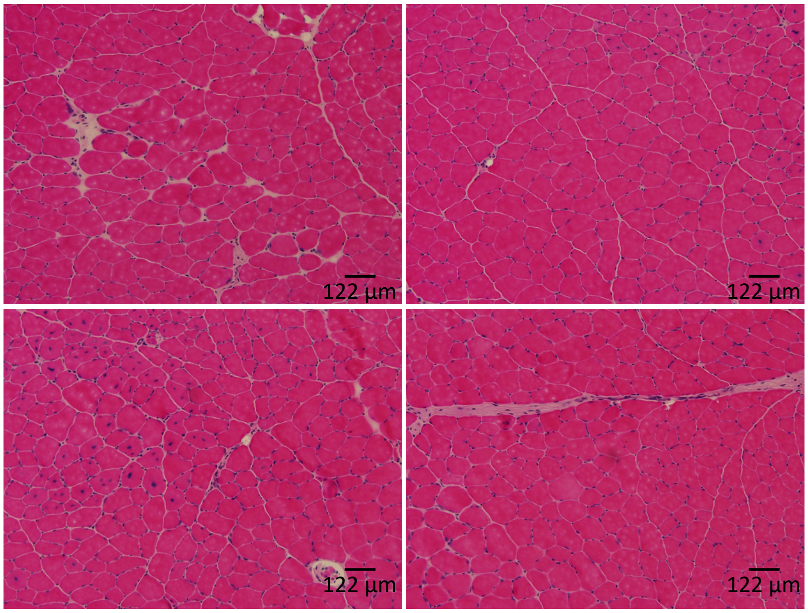

l.

H&E staining: 2Wks in C57BL/6 TA
7167-TA/R: U6.milacZ (3E+09 particles)+U6.miR-675-2 (5E+10 particles)

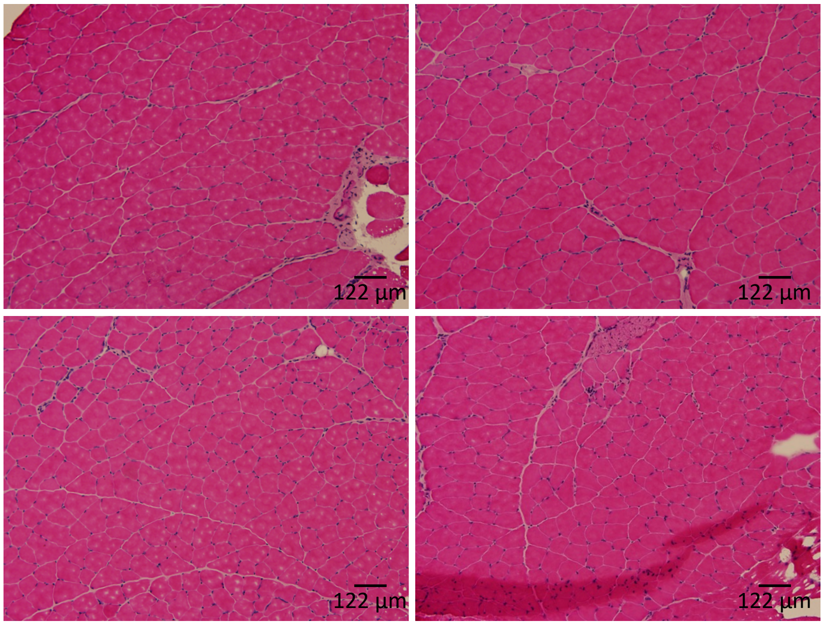

m.

H&E staining: 2Wks in C57BL/6 TA
7252-TA/L: U6.miR-675-2 (5E+10 particles)+DUX4-FL (3E+09 particles)

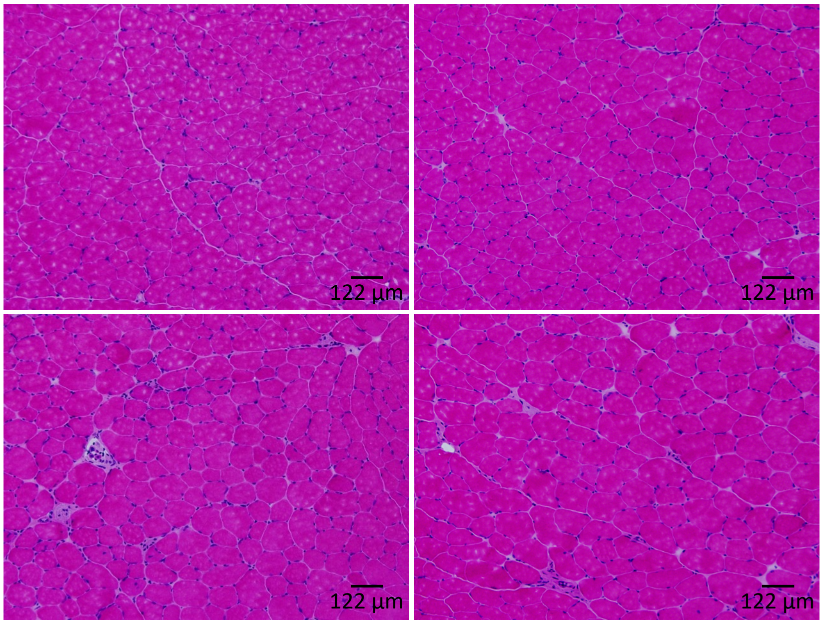

n.

H&E staining: 2Wks in C57BL/6 TA
7252-TA/R: CMV.eGFP (5E+10 particles)+DUX4-FL (3E+09 particles)

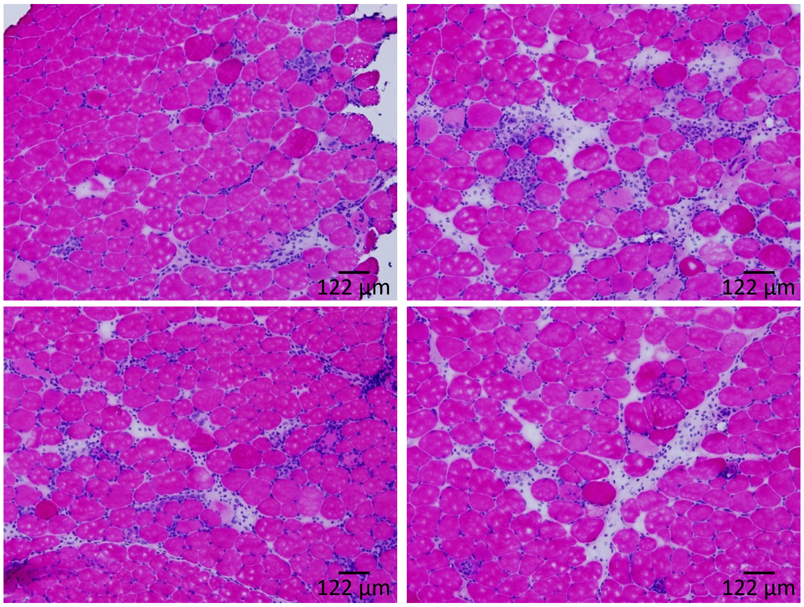

o.

H&E staining: 2Wks in C57BL/6 TA
7253-TA/L: U6.miR-675-2 (5E+10 particles)+DUX4-FL (3E+09 particles)

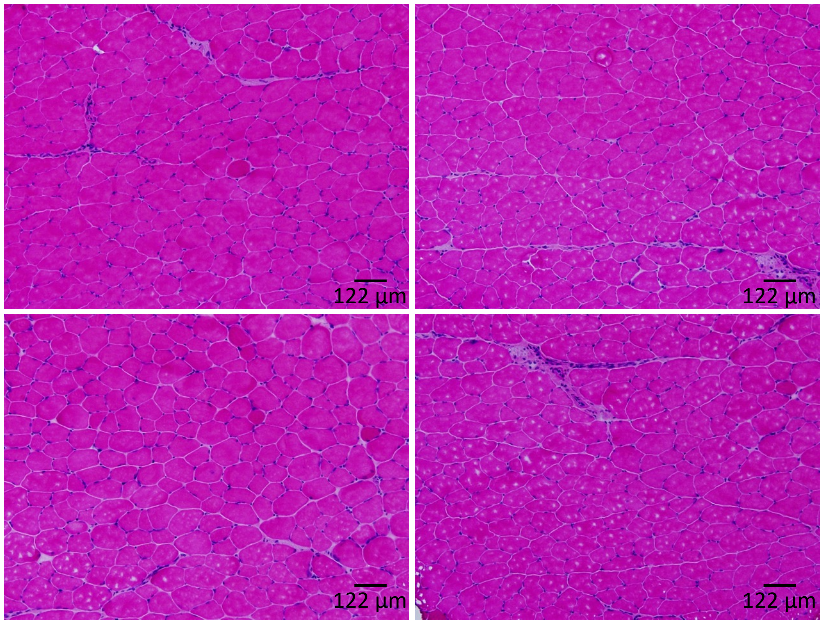

p.

H&E staining: 2Wks in C57BL/6 TA
7253-TA/R: CMV.eGFP (5E+10 particles)+DUX4-FL (3E+09 particles)

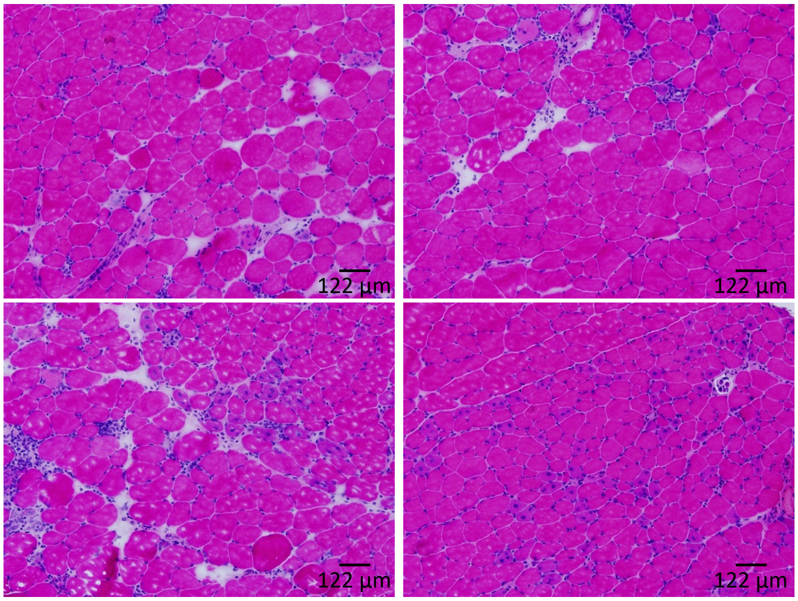

Supplementary Figure 15: a-p. Hematoxylin and eosin (H&E) staining of co-injected Tibialis anterior (TA) with scAAV.U6.MIR675 and AAV6.CMV.DUX4-FL. Compilation of all 10 µm TA muscle sections stained with H&E 2 weeks after intramuscular (IM) injection of C57BL/6 TA

mouse muscles: co-injection of scAAV6.MIR675 ($5x10^{10}$ particles) and AAV.CMV.DUX4-FL ($3x10^9$ particles) (N=8) or negative control (AAV.milacZ or AAV.eGFP; $3x10^9$) (N=3) as well as TA muscles co-injected with negative control ($5x10^{10}$ particles) and AAV.CMV.DUX4-FL ($3x10^9$ particles) (N=5).

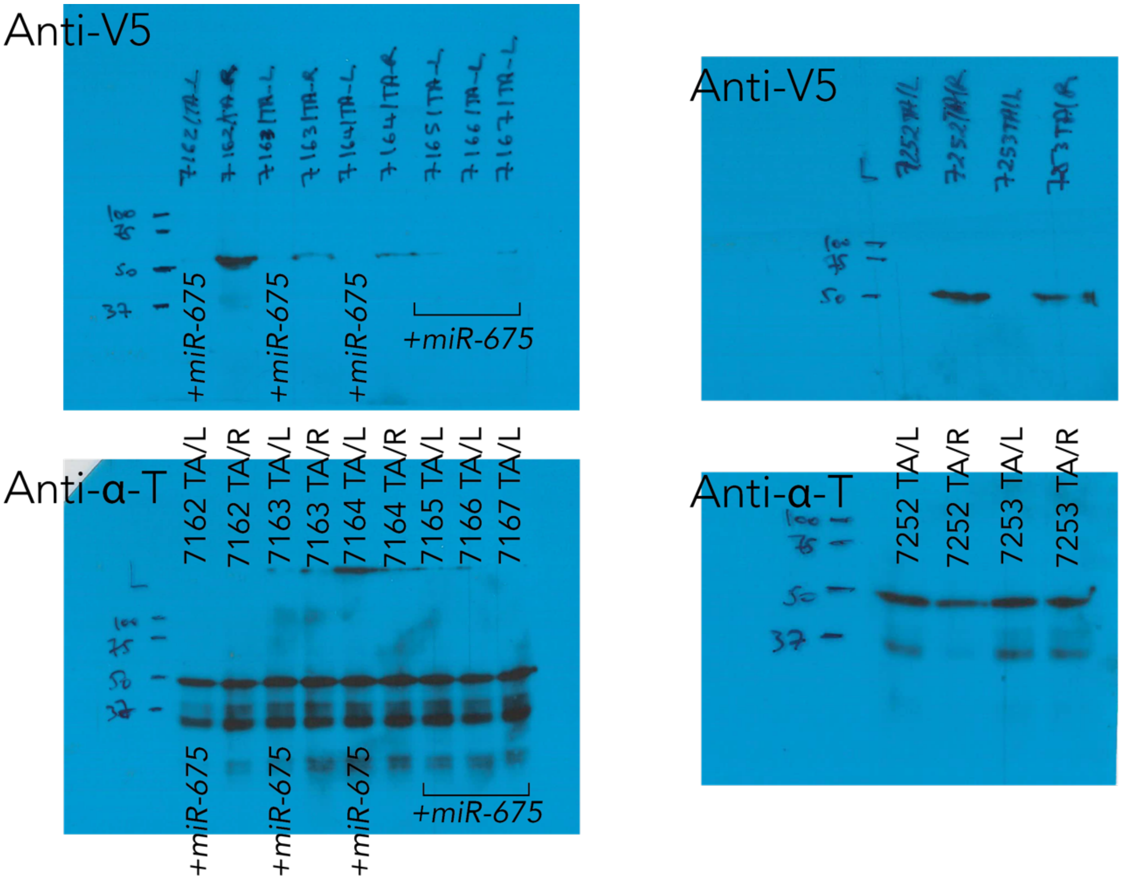

Supplementary Figure 16: Uncropped Western blot from Fig. 8a.

a.

10X

IF staining: 2Wks in C57BL/6 TA
7162-TA/L: U6.miR-675-2 (5E+10 particles)+DUX4-FL (3E+09 particles)

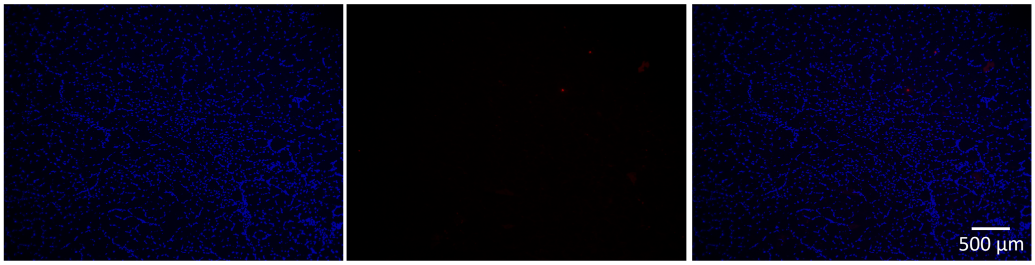

H&E staining: 2Wks in C57BL/6 TA
7162-TA/R: U6.milacZ (5E+10 particles)+DUX4-FL (3E+09 particles)

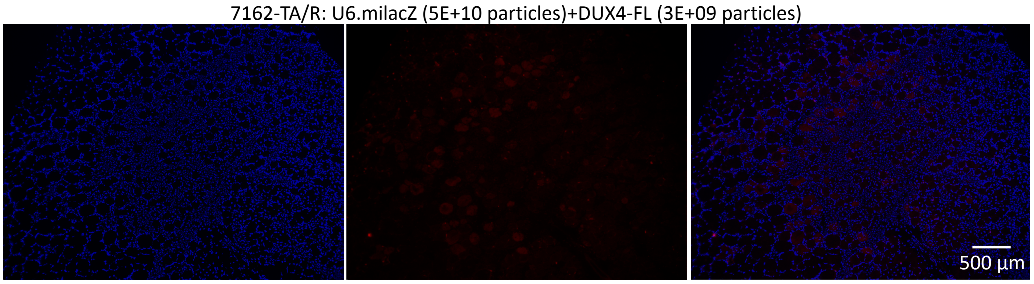

10X

IF staining: 2Wks in C57BL/6 TA
7163-TA/L: U6.miR-675-2 (5E+10 particles)+DUX4-FL (3E+09 particles)

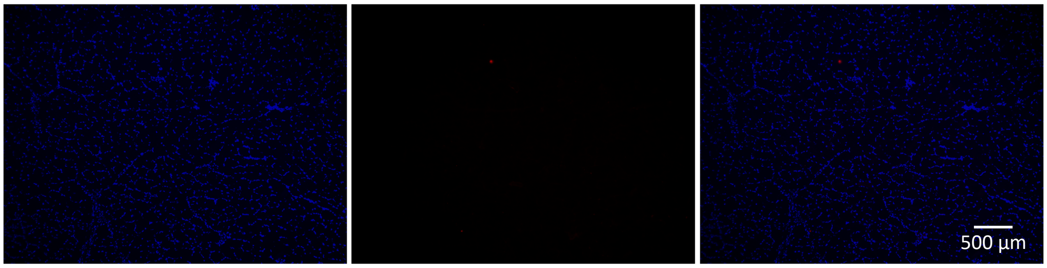

H&E staining: 2Wks in C57BL/6 TA
7163-TA/R: U6.milacZ (5E+10 particles)+DUX4-FL (3E+09 particles)

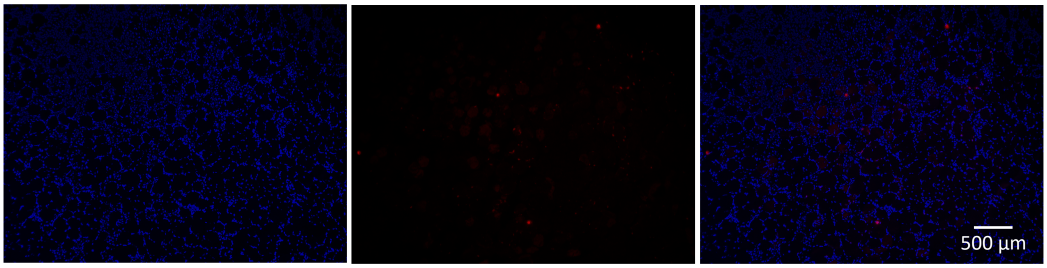

10X

IF staining: 2Wks in C57BL/6 TA
7163-TA/R: U6.milacZ (5E+10 particles)+DUX4-FL (3E+09 particles)

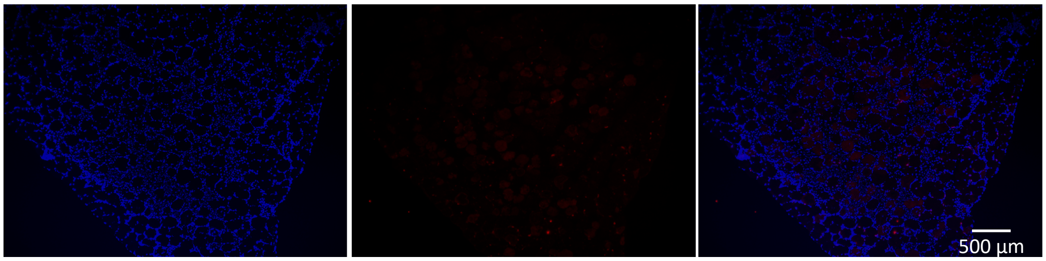

b.

10X

IF staining: 2Wks in C57BL/6 TA
7164-TA/L: U6.miR-675-2 (5E+10 particles)+DUX4-FL (3E+09 particles)

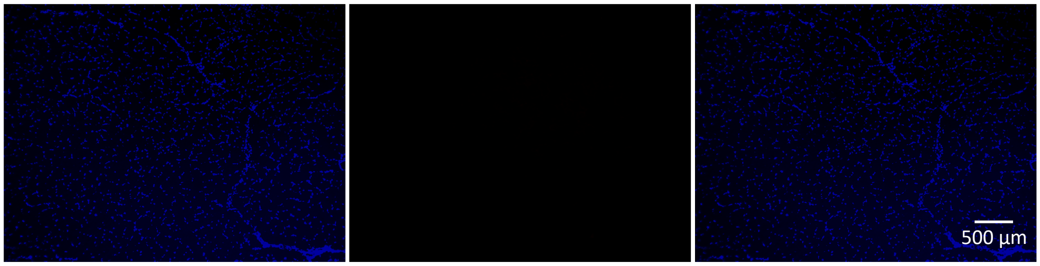

H&E staining: 2Wks in C57BL/6 TA
7164-TA/R: U6.milacZ (5E+10 particles)+DUX4-FL (3E+09 particles)

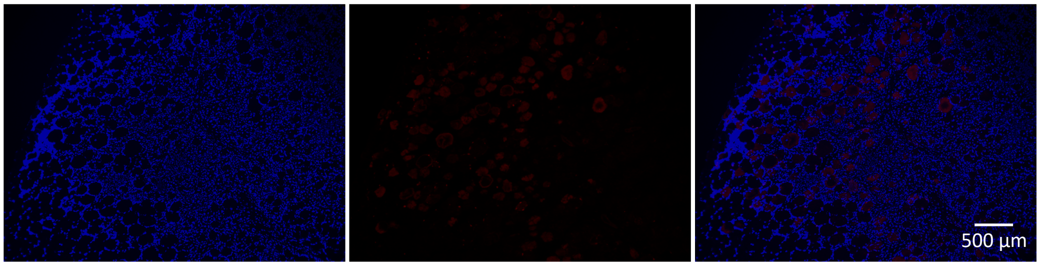

10X

H&E staining: 2Wks in C57BL/6 TA
7164-TA/R: U6.milacZ (5E+10 particles)+DUX4-FL (3E+09 particles)

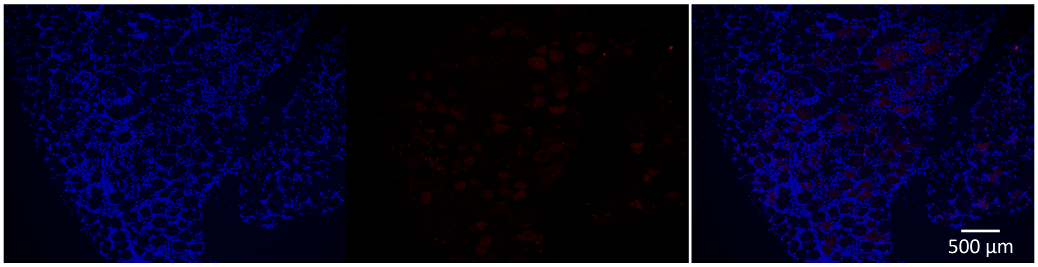

c.

10X

H&E staining: 2Wks in C57BL/6 TA
7165-TA/L: U6.miR-675-2 (5E+10 particles)+DUX4-FL (3E+09 particles)

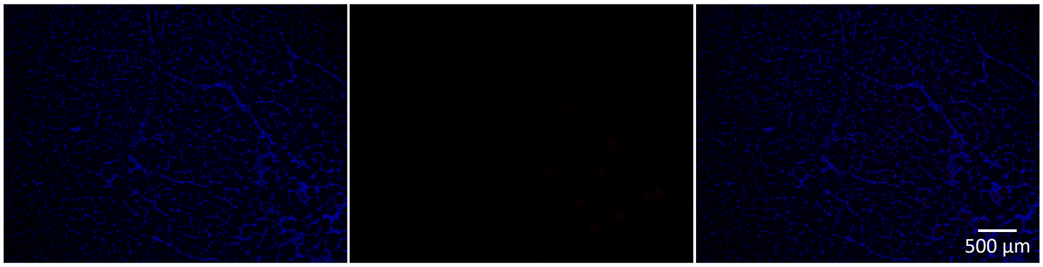

H&E staining: 2Wks in C57BL/6 TA
7165-TA/R: U6.milacZ (3E+09 particles)+U6.miR-675-2 (5E+10 particles)

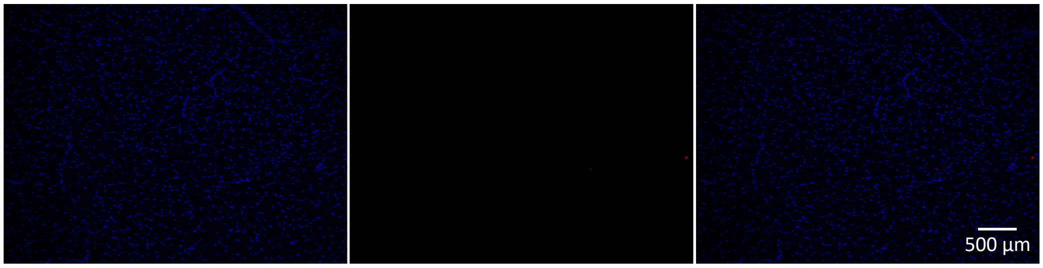

10X

IF staining: 2Wks in C57BL/6 TA
7165-TA/L: U6.miR-675-2 (5E+10 particles)+DUX4-FL (3E+09 particles)

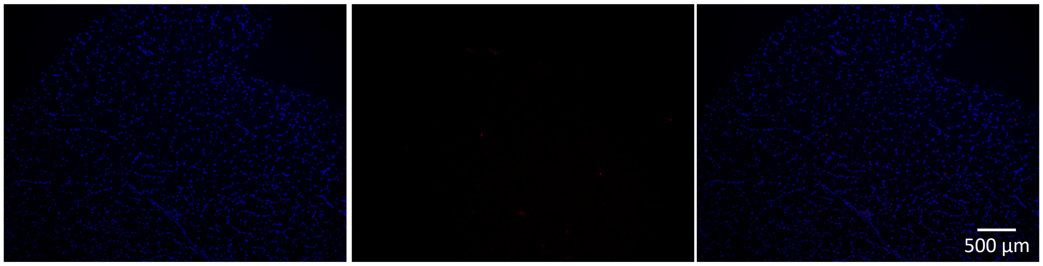

d.

10X

IF staining: 2Wks in C57BL/6 TA
7166-TA/L: U6.miR-675-2 (5E+10 particles)+DUX4-FL (3E+09 particles)

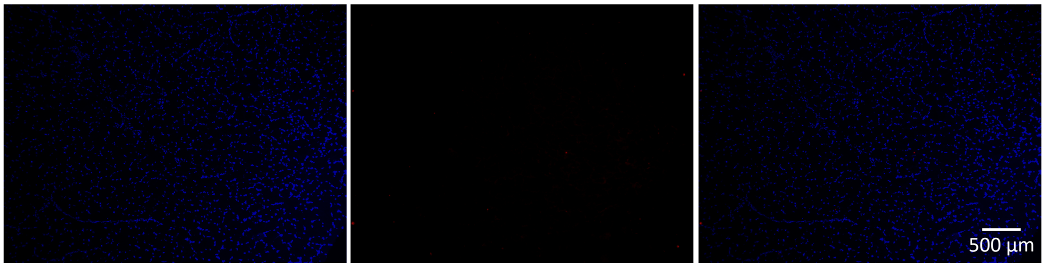

H&E staining: 2Wks in C57BL/6 TA
7166-TA/R: U6.milacZ (3E+09 particles)+U6.miR-675-2 (5E+10 particles)

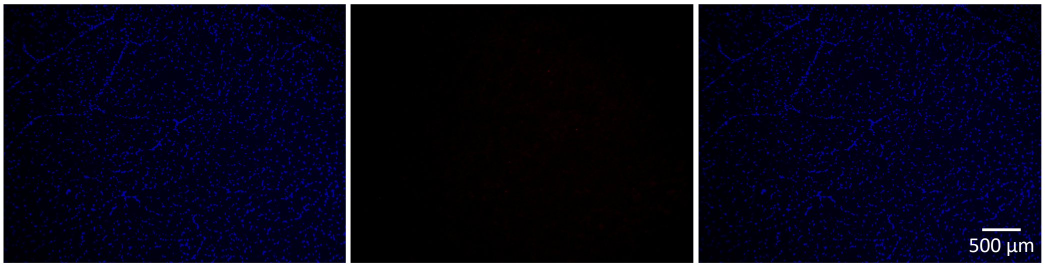

10X

IF staining: 2Wks in C57BL/6 TA
7166-TA/L: U6.miR-675-2 (5E+10 particles)+DUX4-FL (3E+09 particles)

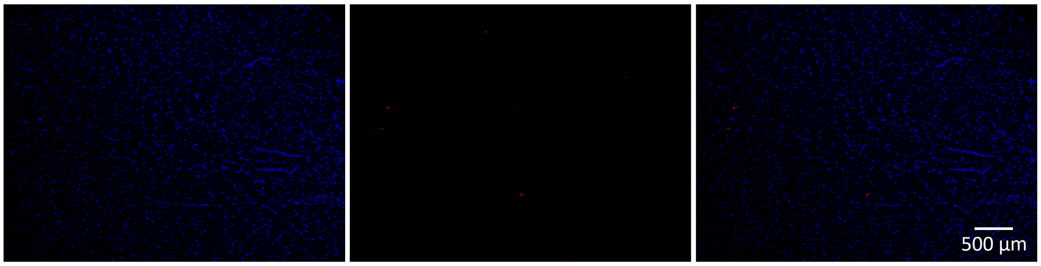

e.

10X

IF staining: 2Wks in C57BL/6 TA
7167-TA/L: U6.miR-675-2 (5E+10 particles)+DUX4-FL (3E+09 particles)

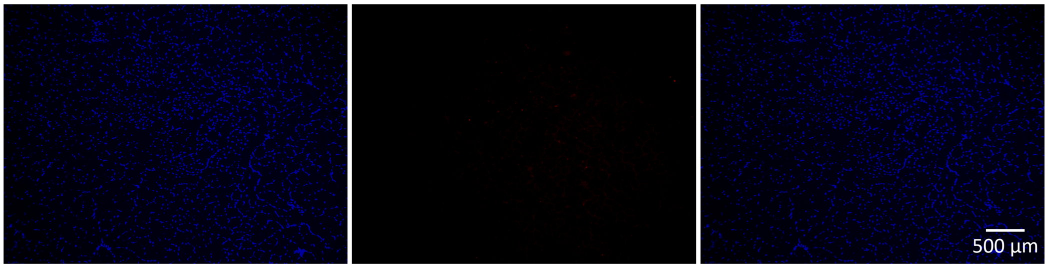

H&E staining: 2Wks in C57BL/6 TA
7167-TA/R: U6.milacZ (3E+09 particles)+U6.miR-675-2 (5E+10 particles)

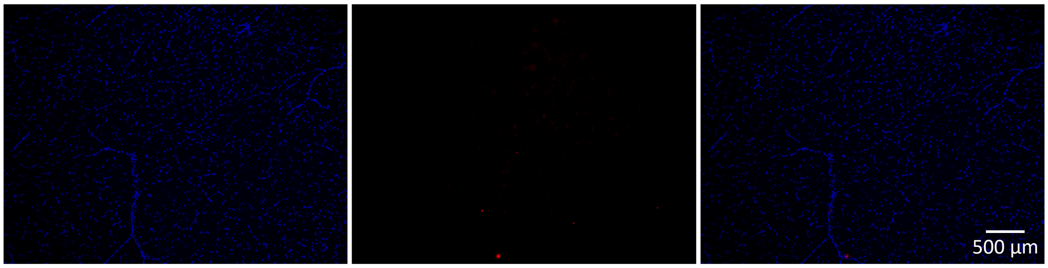

10X

IF staining: 2Wks in C57BL/6 TA
7167-TA/L: U6.miR-675-2 (5E+10 particles)+DUX4-FL (3E+09 particles)

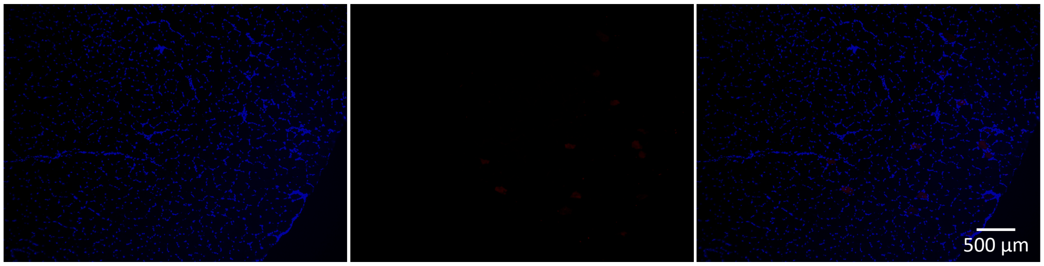

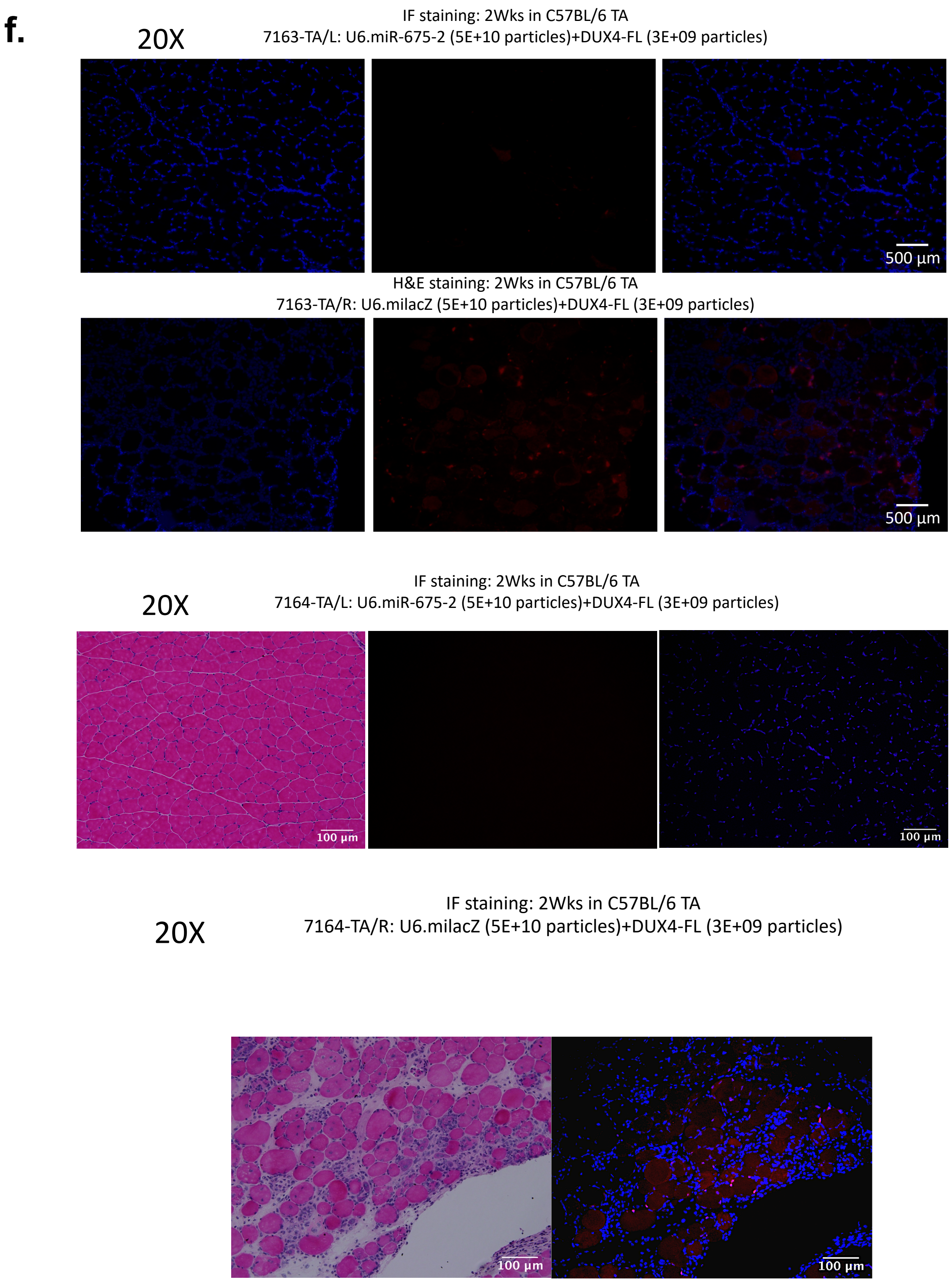

Supplementary Figure 17: a-f. Immunofluorescence (IF) staining of TA muscle sections co-injected with scAAV.U6.MIR675 and AAV6.CMV.DUX4-FL. Immunofluorescence (IF) staining

of 10 μm TA muscle sections 2 weeks after intramuscular (IM) injection of C57BL/6 TA mouse muscles: co-injection of scAAV6.MIR675 ($5x10^{10}$ particles) and AAV.CMV.DUX4-FL ($3x10^9$ particles) (N=6) or AAV.milacZ ($3x10^9$ particles) (N=3) as well as TA muscles co-injected with AAV.milacZ ($5x10^{10}$ particles) and AAV.CMV.DUX4-FL ($3x10^9$ particles) (N=3).

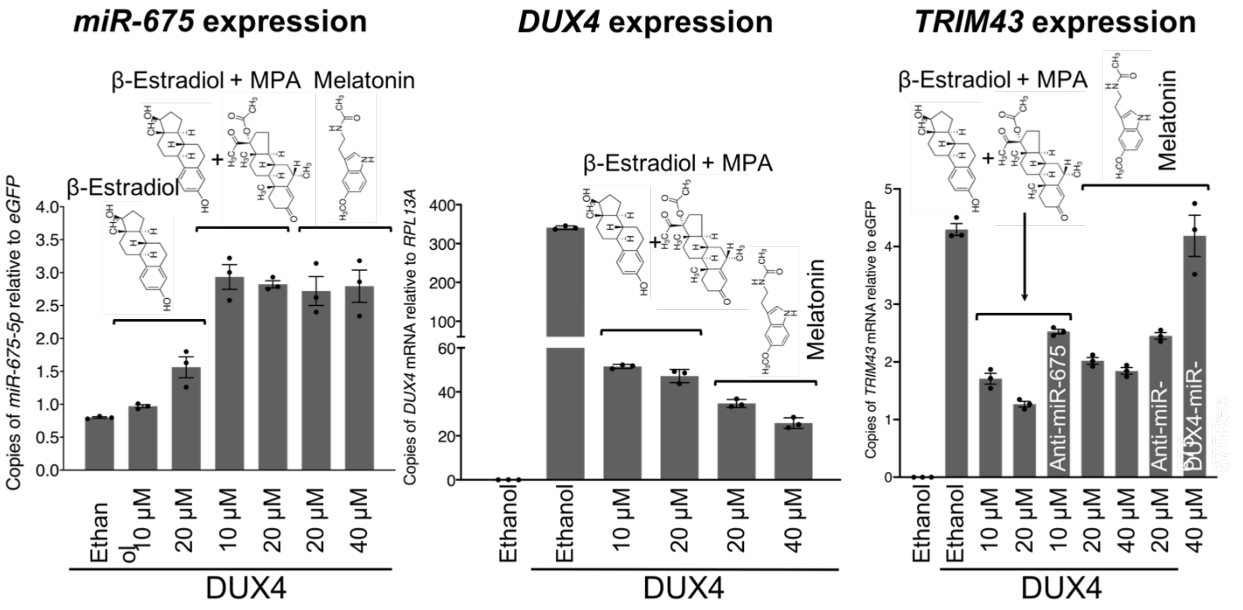

Supplementary Figure 18: β-estradiol, medroxyprogesterone acetate (MPA) and melatonin increased *mir-675* expression and reduced the expression of *DUX4* and *DUX4*-responsive biomarker *TRIM43* in HEK293 cells. *MiR-675-5p*, *DUX4* and *TRIM43* measured by droplet digital PCR (ddPCR). HEK293 cells were transfected with *DUX4* and treated with two drugs individually (i.e. β-estradiol and melatonin) or with a combination of β-estradiol and MPA at 10, 20 or 40 μM at the time of transfection. In one experimental set, cells were also treated with anti-miR-675 or the *miR-675*-resistant *DUX4* expression construct, as additional controls. Results were reported as the average absolute *miR-675-5p*, *DUX4* and *TRIM43* concentration (copies/μL) ± 95% poisson confidence interval normalized to *eGFP* or *hsa-RPL13A*. Data represent mean relative concentration (copies/uL) ± SEM (N=3 independent experiments). One-way ANOVA

followed by Dunnett's multiple comparison tests were performed for statistical analyses. Source data are provided as a Source Data file.

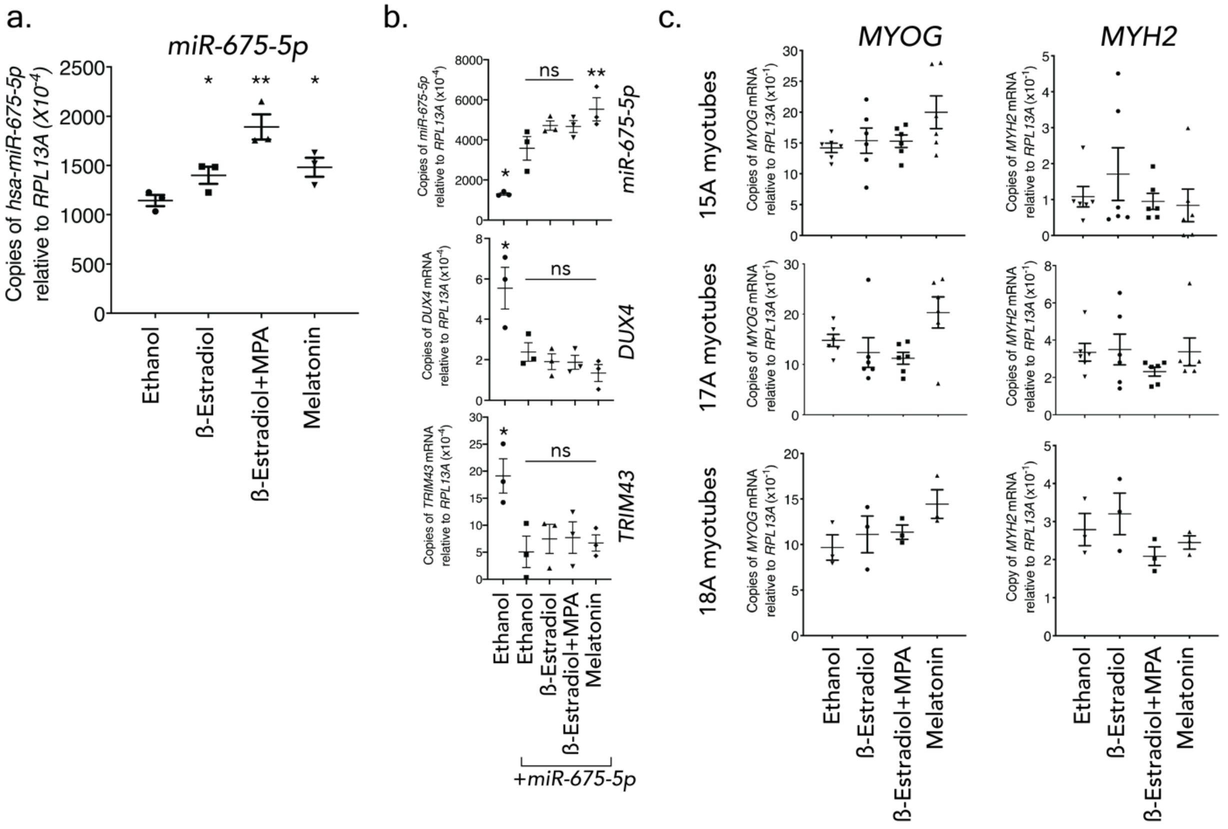

Supplementary Figure 19: Effect of β-estradiol, β-estradiol+medroxyprogesterone acetate (MPA) and melatonin on *TRIM43* and differentiation markers in human FSHD and control myotubes. a. 15V myotubes were treated with 20 μM β-estradiol, medroxyprogesterone acetate (MPA) and melatonin at the 4th day of differentiation, and *miR-675-5p* gene expression was measured 24 hours later. β-estradiol, β-estradiol+MPA and melatonin treatment significantly increased *miR-675-5p* expression by 22±10% (*, P=0.039, N=3), 65±14% (**, P=0.0027, N=3) and 30±11% (*, P=0.026, N=3), respectively. *TRIM43* was undetected in 15V cells, regardless of treatment. b. β-estradiol, β-estradiol+medroxyprogesterone acetate (MPA) and melatonin do not appear to directly inhibit *TRIM43* levels. 15A myotubes were transfected with *miR-675* expression plasmid or negative control miGFP, differentiated for 4 days and then treated with 20 μM of β-

estradiol, β-estradiol and MPA or melatonin. RNA was harvested for ddPCR quantification of *miR-675-5p*, *DUX4* and *TRIM43* expression, and data normalized to controls treated with U6.miGFP and ethanol vehicle. The combination of *miR-675* transfection with and without drug treatments resulted in significantly greater *miR-675-5p* levels compared to controls that received ethanol only (*miR-675* transfection + ethanol, 174±47% increase in *miR-675-5p* levels versus control; *miR-675* + β-estradiol, 261±25%; *miR-675* + β-estradiol and MPA, 257±29%; melatonin, 323±49%; for *miR-675* + ethanol, *, P=0.0092; for β-estradiol-treated, *, P=0.0005; for β-estradiol+MPA-treated, *, P=0.0006 and for melatonin-treated, *, P<0.0001; N=3 independent experiments). Consistent with results throughout this study, increased *miR-675-5p* levels inversely correlated with *DUX4* and *TRIM43* expression. Compared to controls treated with ethanol only, *miR-675* transfected cells showed significantly decreased *DUX4* expression (*miR-675* transfection + ethanol, 57±11% decrease in *DUX4* versus control; *miR-675* + β-estradiol, 65±9% *DUX4* decrease; *miR-675* + β-estradiol and MPA 66±9% decrease, and *miR-675* + melatonin, 76±9% decrease; for *miR-675* + ethanol, P=0.011; for β-estradiol-treated, *, P=0.0045; for β-estradiol+MPA-treated, *, P=0.0043 and for melatonin-treated, *, P=0.0016; N=3 independent experiments). Similarly, increased *miR-675-5p* levels inversely correlated with significantly reduced TRIM43 expression (*miR-675* transfection + ethanol, 73±16 % decrease in *TRIM43* levels versus control; *miR-675* + β-estradiol, 61±15%; *miR-675* + β-estradiol and MPA, 60±17%; melatonin, 65±10%; for *miR-675* + ethanol, *, P=0.0139; for β-estradiol-treated, *, P=0.0384; for β-estradiol+MPA-treated, *, P=0.0426 and for melatonin-treated, *, P=0.0279; N=3 independent experiments).

Importantly, although there was a trend toward higher *miR-675-5p* levels in cells that received transfected *miR-675* plus drugs but values were not significantly (ns) different from cells transfected with *miR-675* and treated with ethanol, except for the *miR-675* + melatonin treated cells (**, P=0.0228; for β-estradiol-treated, ns, P=0.2164; for β-estradiol+MPA-treated, ns, P=0.2452; N=3 independent experiments). Similarly, *DUX4* and *TRIM43* inhibition was not

significantly different among the 4 groups transfected with *miR-675*, regardless of additional drug or vehicle treatment. These results suggested that the drug treatments did not directly inhibit *TRIM43*. Results were reported as the average absolute *miR-675-5p*, *DUX4* and *TRIM43* concentration (copies/µL) ± 95% poisson confidence interval normalized to *hsa-RPL13A*. **c.** β-estradiol, β-estradiol + medroxyprogesterone acetate (MPA) and melatonin do not significantly impact expression of muscle differentiation markers in 15A, 17A and 18A myoblasts/myotubes. Cells were treated with 20 µM β-estradiol, β-estradiol + medroxyprogesterone acetate (MPA) or melatonin at the 4th day of differentiation, and *MYOG* and *MYH2* gene expression were measured 24 hours later. In all cell lines and in all treatment conditions, no significant change in *MYOG* or *MYH2* was measured. For **a.**, **b.** and **c.**, results were reported as the average absolute *miR-675-5p*, *DUX4, TRIM43, MYOG* and *MYH2* concentration (copies/µL) ± 95% poisson confidence interval normalized to *hsa-RPL13A*. Data represent mean relative concentration (copies/uL) ± SEM (N=3 or 6 independent experiments). One-way ANOVA followed by Dunnett's multiple comparison tests were performed for statistical analyses. Source data are provided as a Source Data file.

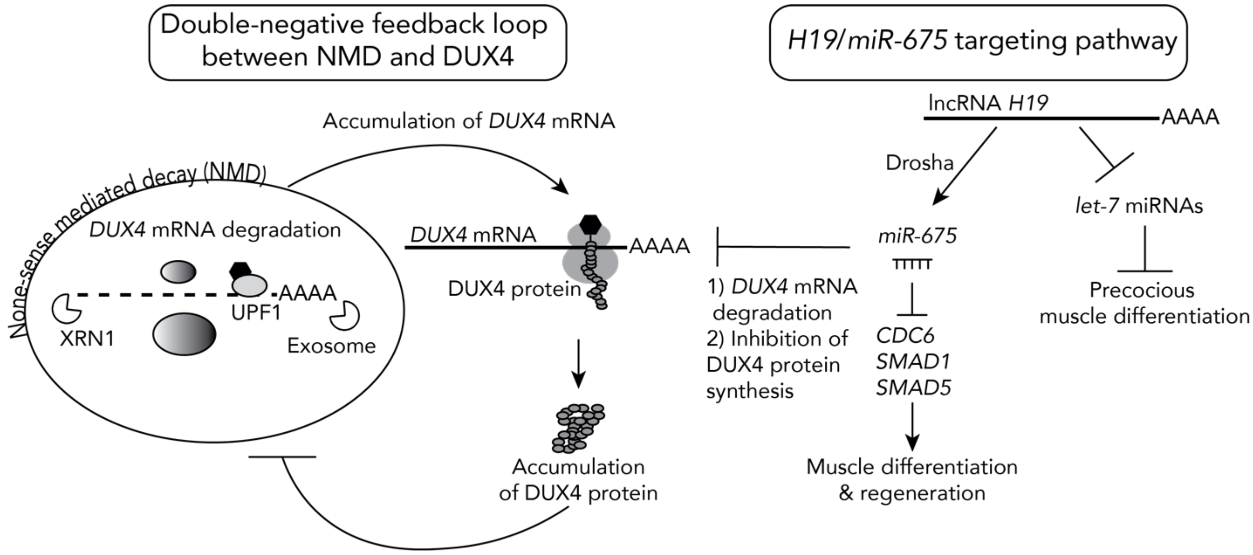

Supplementary Figure 20: *DUX4* mRNA is subject to regulation by non-sense-mediated decay (NMD) and RNA interference (RNAi). *DUX4* mRNA is subject to degradation by the non-sense mediated decay (NMD) pathway. In instances where *DUX4* mRNA evades NMD and become polyadenylated and exported for translation, it could be bound by *miR-675-5p*, thereby triggering RNAi against the *DUX4* mRNA in the cytoplasm. As such, we propose that increasing *miR-675* levels, using drugs or gene therapy, could offer a treatment for FSHD by decreasing *DUX4*. In addition, this approach could have added benefits to muscle: *miR-675*, which is derived from the *H19* long non-coding RNA (lncRNA), also inhibits the anti-differentiation *SMAD1* and *SMAD5* transcription factors and the DNA replication initiation factor *CDC6*, promoting muscle differentiation and regeneration. In addition, *H19* acts as a sponge to the *let-7* family of miRNAs and prevents precocious muscle differentiation. Thus, in addition to inhibiting *DUX4*, the *miR-675*/*H19* axis could have an added benefit to FSHD by helping to also counteract differentiation defects that have been previously reported in FSHD muscle cells.

Supplementary Table 1: Fold-Change of gene expression in myotubes (MT) differentiated for 4 days versus myoblasts (Supplementary Fig. 10).

		**Fold-Change (MT vs MB)**	**P-value**	**N**
**15V Ctrl**	***H19***	89±3	P=0.0048, ANOVA	3
	pri-mir-675	2.3±0.1	P<0.0001, ANOVA	3
	miR-675-5p	2.2±0.1	P<0.0001, ANOVA	3
	miR-675-3p	7.7±1.0	P<0.0001, ANOVA	3
**15A FSHD**	***H19***	433±23	P<0.0001, ANOVA	3
	pri-mir-675	2.3±0.2	P<0.0001, ANOVA	3
	miR-675-5p	1.8±0.1	P<0.0001, ANOVA	3
	miR-675-3p	8.0±0.2	P<0.0001, ANOVA	3

**17A FSHD**	***H19***	158±10	P=0.0016, ANOVA	3
	pri-mir-675	3.3±0.4	P<0.0001, ANOVA	3
	miR-675-5p	1.3±0.1	P<0.0001, ANOVA	3
	miR-675-3p	1.4±0.1	P<0.0001, ANOVA	3

Supplementary Table 2: Percent fold-change of gene expression (*miR-675-5p*, *DUX4* and *TRIM43*) in 5-days differentiated 15A, 17A and 18A myotubes following treatment with β-estradiol, β-estradiol+MPA or melatonin (Fig. 8). NS: not significant. I.E.: independent experiments (N). 5DD: 5-days differentiated. Results were reported as the average absolute *DUX4, miR-675-5p or TRIM43* concentration (copies/μL) ± 95% poisson confidence interval normalized to *hsa-RPL13A*. Data represent mean relative concentration (copies/uL) ± SEM (N=3 independent experiments). ANOVA statistical tests were performed on data from individual experiments.

	Concentration (μM)	Cell lines 5DD myotubes	***miR-675-5p*** (% increase)	***DUX4*** (% decrease)	***TRIM43*** (% decrease)	***N***
β-estradiol	20	15A	47±11 | *, P=0.025	70±38 | *, P=0.018	46±40 | NS, P=0.23	N=6 I.E.
		17A	38±09 | *, P=0.0275	63±19 | *, P=0.0103	31±12 | *, P=0.0377	N=3 I.E.
		18A	382±44 | ***, P=0.0003	49±19 | *, P=0.0143	57±07 | **, P=0.0024	N=3 I.E.
β-estradiol + MPA	20 each	15A	52±15 | *, P=0.012	86±39 | **, P=0.0037	74±45 | *, P=0.0296	N=6 I.E.
		17A	50±15 | **, P=0.0060	51±11 | *, P=0.0298	65±15 | ***, P=0.0006	N=3 I.E.
		18A	258±49 | **, P=0.0032	81±20 | ***, P=0.0007	84±09 | ***, P=0.0002	N=3 I.E.
Melatonin	20	15A	44±16 | *, P=0.035	88±40 | **, P=0.0030	75±45 | *, P=0.0271	N=6 I.E.

		17A	48±09 | **, P=0.0079	55±09 | *, P=0.0212	30±12 | *, P=0.0446	N=3 I.E.
		18A	154±59 | *, P=0.0476	38±17 | *, P=0.0470	70±15 | ***, P=0.0006	N=3 I.E.

Supplementary Table 3: Complete list of all primers used in this study.

CMV.DUX4 miR-675Res construct	forward: 5’ CCGAGAATTCCTCGACTTATTAATAGTAATCAATTACGGGGTCA 3’<br>forward middle: 5’ ACCCAAGATCTGGGGCAAGGTGGGCAAAAGCCGGGAGGA 3’<br>reverse middle: 5’ CACCTTGCCCCAGATCTTGGGTGCCTGAGGGTGGGAGAG 3’,<br>reverse: 5’ CGGGTACCCTACGTAGAATCGAGCCCGAGGAG 3’
CMV.DUX4-FL/CMV.eGFP and CMV.eGFP cloned into CMV.DUX4-miR-675Res	forward: TTACTAGTATTAATAGTAATCAATTACGG<br>reverse: CAATGA ATTCGTTAATGATTAACCCGCCAT
RenLuc-DUX4-FL expression plasmid	forward: 5’ CCGGCTCGAGATGGCCCTCCCGACAC 3’<br>reverse: 5’ ACGACTAGTGGGAGGGGGCATTTTAATATATCTC 3’
RenLuc-DUX4 ORF-miR-675Res expression plasmid	forward: 5’ CCGGCTCGAGATGGCCCTCCCGACAC 3’<br>forward middle: 5’ CGGGCAAAAGCCGGGAGGA 3’<br>reverse middle: 5’ TCCTCCCGGCTTTTGCCCGGCCTGAGGGTGGGAGA 3’<br>reverse: 5’ AGCGGCCGCAAGCTCCTCCAGCAGAGC 3’

Supplementary Powerpoint P1: H&E staining of 10 µm muscle sections from C57BL/6 TA mouse muscles injected with scAAV6.MIR675 construct expressing H1.MIR675 ($5x10^{10}$ particles) show toxicity (N=3).

Supplementary Powerpoint P2: Compilation of all 10 µm TA muscle sections stained with H&E 2 weeks after intramuscular (IM) injection of C57BL/6 TA mouse muscles: co-injection of scAAV6.MIR675 ($5x10^{10}$ particles) and AAV.CMV.DUX4-FL ($3x10^{9}$ particles) (N=8) or negative control (AAV.milacZ or AAV.eGFP; $3x10^{9}$) (N=3) as well as TA muscles co-injected with negative control ($5x10^{10}$ particles) and AAV.CMV.DUX4-FL ($3x10^{9}$ particles) (N=5).

Supplementary Powerpoint P3: Immunofluorescence (IF) staining of 10 µm TA muscle sections 2 weeks after intramuscular (IM) injection of C57BL/6 TA mouse muscles: co-injection of scAAV6.MIR675 ($5x10^{10}$ particles) and AAV.CMV.DUX4-FL ($3x10^{9}$ particles) (N=6) or AAV.milacZ ($3x10^{9}$ particles) (N=3) as well as TA muscles co-injected with AAV.milacZ ($5x10^{10}$ particles) and AAV.CMV.DUX4-FL ($3x10^{9}$ particles) (N=3).

References

1. S. Griffiths-Jones, R. J. Grocock, S. van Dongen, A. Bateman and A. J. Enright, miRBase: microRNA sequences, targets and gene nomenclature. Nucleic Acids Res 34, D140-144 (2006).
2. M. Kertesz, N. Iovino, U. Unnerstall, U. Gaul and E. Segal, The role of site accessibility in microRNA target recognition. Nat Genet 39, 1278-1284 (2007).
3. L. M. Wallace, J. Liu, J. S. Domire, S. E. Garwick-Coppens, S. M. Guckes, J. R. Mendell, K. M. Flanigan and S. Q. Harper, RNA interference inhibits DUX4-induced muscle toxicity in vivo: implications for a targeted FSHD therapy. Mol Ther 20, 1417-1423 (2012).
4. B. K. Dey, K. Pfeifer and A. Dutta, The H19 long noncoding RNA gives rise to microRNAs miR-675-3p and miR-675-5p to promote skeletal muscle differentiation and regeneration. Genes Dev 28, 491-501 (2014).
5. Cai, X. & Cullen, B. R. The imprinted H19 noncoding RNA is a primary microRNA precursor. *RNA* **13**, 313-316, (2007).
6. L. M. Wallace, N. Y. Saad, N. K. Pyne, A. M. Fowler, J. O. Eidahl, J. S. Domire, D. A. Griffin, A. C. Herman, Z. Sahenk, L. R. Rodino-Klapac and S. Q. Harper, Pre-clinical Safety and Off-Target Studies to Support Translation of AAV-Mediated RNAi Therapy for FSHD. Mol Ther Methods Clin Dev 8, 121-130 (2018).
