# Supplementary material for: Human miRNA miR-675 inhibits DUX4 expression and may be exploited as a potential treatment for Facioscapulohumeral muscular dystrophy
Source: Nat Commun. 2021 Dec 8;12:7128. doi: 10.1038/s41467-021-27430-1 (PMC8654987; doi:10.1038/s41467-021-27430-1)
Supplement: Supplementary file 2 — Reporting Summary [file 41467_2021_27430_MOESM2_ESM.pdf]

nature research

Corresponding author(s): Scott Q. Harper
Last updated by author(s): 11/16/2021

Reporting Summary

Nature Research wishes to improve the reproducibility of the work that we publish. This form provides structure for consistency and transparency in reporting. For further information on Nature Research policies, see our Editorial Policies and the Editorial Policy Checklist.

Statistics

For all statistical analyses, confirm that the following items are present in the figure legend, table legend, main text, or Methods section.

n/a	Confirmed	
☐	☒	The exact sample size (*n*) for each experimental group/condition, given as a discrete number and unit of measurement
☐	☒	A statement on whether measurements were taken from distinct samples or whether the same sample was measured repeatedly
☐	☒	The statistical test(s) used AND whether they are one- or two-sided<br>*Only common tests should be described solely by name; describe more complex techniques in the Methods section.*
☒	☐	A description of all covariates tested
☒	☐	A description of any assumptions or corrections, such as tests of normality and adjustment for multiple comparisons
☐	☒	A full description of the statistical parameters including central tendency (e.g. means) or other basic estimates (e.g. regression coefficient) AND variation (e.g. standard deviation) or associated estimates of uncertainty (e.g. confidence intervals)
☐	☒	For null hypothesis testing, the test statistic (e.g. *F*, *t*, *r*) with confidence intervals, effect sizes, degrees of freedom and *P* value noted<br>*Give P values as exact values whenever suitable.*
☒	☐	For Bayesian analysis, information on the choice of priors and Markov chain Monte Carlo settings
☒	☐	For hierarchical and complex designs, identification of the appropriate level for tests and full reporting of outcomes
☒	☐	Estimates of effect sizes (e.g. Cohen's *d*, Pearson's *r*), indicating how they were calculated

Our web collection on statistics for biologists contains articles on many of the points above.

Software and code

Policy information about availability of computer code

Data collection	For luciferase assay, data was collected using the GloMax 96, microplate luminometer.<br>For quantitative RT-PCR, data was collected using the Bio Rad CFX96 Real-time system.<br>For ddPCR, we generated droplets using the Automatic Droplet generator QX200 AutoDG (Bio-Rad), and amplified the reactions in a C1000 Touch™ Thermal Cycler with 96–Deep Well Reaction Module (Bio-Rad). We read droplets using the QX200 droplet reader (Bio-Rad).<br>Images of cell cultures and Skeletal muscle sections were taken using the Olympus BX61 microscope and the Olympus cellSens standard 1.9 software.
Data analysis	Data was analyzed using the Graphpad Prism 8 software.<br>Comet assay data was analyzed using the Tritek CometScore software 2.0.<br>For ddPCR, we analyzed data using the QuantaSoft analysis software 1.0 (Bio-Rad).<br>For flow cytometry, we analyzed data using the FlowJo 10.7.1 software.

For manuscripts utilizing custom algorithms or software that are central to the research but not yet described in published literature, software must be made available to editors and reviewers. We strongly encourage code deposition in a community repository (e.g. GitHub). See the Nature Research guidelines for submitting code & software for further information.

Data

Policy information about availability of data

All manuscripts must include a data availability statement. This statement should provide the following information, where applicable:

- Accession codes, unique identifiers, or web links for publicly available datasets
- A list of figures that have associated raw data
- A description of any restrictions on data availability

miRbase database can be found on www.miRbase.com.
PITA target prediction algorithm can be found on https://genie.weizmann.ac.il/pubs/mir07/mir07_prediction.html.

Field-specific reporting

Please select the one below that is the best fit for your research. If you are not sure, read the appropriate sections before making your selection.

☒ Life sciences ☐ Behavioural & social sciences ☐ Ecological, evolutionary & environmental sciences

For a reference copy of the document with all sections, see nature.com/documents/nr-reporting-summary-flat.pdf

Life sciences study design

All studies must disclose on these points even when the disclosure is negative.

Sample size	The objective of the study was to explore new strategies for the treatment of FSHD. FSHD is caused by de-repression of the DUX4 gene, which is toxic to muscle. FSHD therapies are thus focused on inhibiting DUX4, which was our main goal in this paper. However, instead of using conventional approaches (i.e antisense oligos (ASO), siRNAs, shRNA or artificial miRNAs), here we decided to test a novel strategy to direct RNAi against DUX4. Specifically, we reasoned that we could use drugs to up-regulate endogenous human microRNAs that naturally direct RNAi against DUX4, and that this would offer a novel strategy to inhibit the gene with RNAi. To our knowledge, this has never been done before for any dominant genetic disease. In this study, we show that mir-675 inhibits DUX4 efficiently and reduces DUX4-associated phenotypes in human HEK293 cells and FSHD muscle cell lines. We also show that mir-675 functions within a gene therapy vector to inhibit DUX4-associated pathologies in vivo – in an AAV.DUX4 mouse model we previously developed and published (PMID: 21446026). In the small molecule treatment assay, we used three different FSHD cell lines. For the 15A FSHD cell line, we performed 6 independent experiments. For the 17A and 18A FSHD cell lines, we performed 3 independent experiments. For all in vitro experiments, no sample size calculation was performed. Sample size was determined based on our previously published studies (PMID: 21446026; PMID: 22508491; PMID: 29387734). Based on our aforementioned previously published studies, these sample sizes were found to be sufficient.<br>For the in vivo study, sample size was chosen based on our previously published studies (PMID: 22508491; PMID: 29387734).
Data exclusions	No data were excluded from the analyses.
Replication	For the in vitro study, we performed between N=3 to N=6 independent experiments depending on the assay. We performed 6 independent blinded western blots when testing mir-675 specific inhibition of DUX4 expression. All raw western blot images are included in the submission. In the small molecule treatment assay, we used three different FSHD cell lines. For the 15A FSHD cell line, we performed 6 independent experiments. For the 17A and 18A FSHD cell lines, we performed 3 independent experiments. For the in vivo study, sample size was chosen based on our previously published studies (PMID: 22508491; PMID: 29387734). All attempts at replication were successful.
Randomization	For the in vitro work, independent experiments were randomized by choosing different days, and for transfection of cells, by transfecting the different samples with random order every time. The blinded western blot experiments also helped with the randomization of the experiment. For the in vivo work, AAV vectors were injected in tibialis anterior muscles of randomly selected mice. Every mouse received a co-injection and the contralateral control were distributed evenly across randomly assigned wild-type mice.
Blinding	We performed 6 independent blinded western blots when testing mir-675 specific inhibition of DUX4 expression. When we performed blinded western blots, we asked a lab member who is not involved in the study to blind the DUX4 and miRNA expression plasmids before we transfect them into HEK293 cells. We also performed the SDS-PAGE gel and immunoblotting under blinding conditions. At the end of every experiment, the same lab member unblinded the blots. For all other experiments, blinding was not required as all data was collected by objective measurements.

Reporting for specific materials, systems and methods

We require information from authors about some types of materials, experimental systems and methods used in many studies. Here, indicate whether each material, system or method listed is relevant to your study. If you are not sure if a list item applies to your research, read the appropriate section before selecting a response.

Materials & experimental systems

n/a	Involved in the study
	☒ Antibodies
	☒ Eukaryotic cell lines
☒	Palaeontology and archaeology
	☒ Animals and other organisms
	☒ Human research participants
☒	Clinical data
☒	Dual use research of concern

Methods

n/a	Involved in the study
☒	ChIP-seq
☒	Flow cytometry
☒	MRI-based neuroimaging

Antibodies

Antibodies used

1) Mouse monoclonal antibody to V5 (Invitrogen; catalog# R961-25; horseradish peroxidase [HRP]-coupled, lot# GR3224488-1).
2) Rabbit polyclonal eGFP antibody (Abcam; catalog# ab290; lot# 841067).
3) HRP-coupled goat anti-rabbit secondary antibody (Jackson ImmunoResearch; catalog# 115-035-144) .
4) Cdc6 rabbit monoclonal antibody (Cell signaling technology; catalog# C42F7; clone# 3387S; lot# 2).
5) α-tubulin rabbit polyclonal antibody (Abcam; catalog# ab15246; lot# GR322829-1).
6) B-actin monoclonal antibody produced in mouse (Sigma-Aldrich, catalog# A5441, clone# AC-15; lot# 0000110619).
7) DUX4 mouse monoclonal antibody (Millipore Sigma; Catalog# MABD116; clone# mAB 9A12; lot# 3587270).

Validation

All primary antibodies were validated by the manufacturer.
1) Mouse monoclonal antibody to V5; validated for ELISA and western blots. https://www.thermofisher.com/antibody/product/V5-Tag-Antibody-Monoclonal/R961-25.

2) Rabbit polyclonal eGFP antibody (ab290; Abcam); suitable for Flow Cyt, ELISA, ICC/IF, ChIP, IHC-FrFl, ChIP/Chip, IHC - Wholemount, Electron Microscopy, IHC-FoFr, ICC, IHC-P, IHC-Fr, IP, WB. https://www.abcam.com/gfp-antibody-chip-grade-ab290.html.

3) HRP-coupled goat anti-rabbit secondary antibody (115-035-144; Jackson ImmunoResearch); suitable for WB.

4) Cdc6 rabbit monoclonal antibody (C42F7; Cell signaling technology); suitable for W-Western IP-Immunoprecipitation IHC-Immunohistochemistry ChIP-Chromatin Immunoprecipitation IF-Immunofluorescence F-Flow Cytometry E-P-ELISA-Peptide. https://www.cellsignal.com/products/primary-antibodies/cdc6-c42f7-rabbit-mab/3387.

5) α-tubulin rabbit polyclonal antibody (ab15246; Abcam); suitable for: ICC/IF, IHC-Fr, WB, IHC-P, IP. https://www.abcam.com/alpha-tubulin-antibody-microtubule-marker-ab15246.html.

6) B-actin monoclonal antibody produced in mouse (Sigma-Aldrich, A5441, clone# AC-15); application(s): immunohistochemistry (formalin-fixed, paraffin-embedded sections): suitable, indirect ELISA: suitable, indirect immunofluorescence: 1:1,000-1:2,000 using cultured human or chicken fibroblasts. western blot: 1:5,000-1:10,000 using cultured human or chicken fibroblast cell extracts. https://www.sigmaaldrich.com/US/en/product/sigma/a5441?context=product.

7) DUX4 mouse monoclonal antibody (Millipore Sigma; Catalog# MABD116; clone# mAB 9A12); Detect Dux4 using this mouse monoclonal antibody, Anti-Dux4 Antibody, clone 9A12 validated for use in western blotting, ICC, Electrophoretic Mobility Shift Assay & IP. Key applications: Western Blotting, Immunocytochemistry, Electrophoretic Mobility Shift Assay, Immunoprecipitation. Application Notes: This antibody has been shown to work in immunocytochemistry applications (Dixit et al. 2007). This antibody has been shown to work in electrophoretic mobility shift assays (Dixit et al. 2007). This antibody has been shown to work in immunoprecipitation applications (Anseau et al., 2009). https://www.emdmillipore.com/US/en/product/Anti-Dux4-Antibody-clone-9A12,MM_NF-MABD116?ReferrerURL=https%3A%2F%2Fwww.google.com%2F

Eukaryotic cell lines

Policy information about cell lines

Cell line source(s)

HEK293 cells from ATCC [HEK-293 (ATCC® CRL-1573™)]

The following human muscle cell lines were used in this study: 15A, 17A and 18A FSHD human myoblasts as well as 15V control human myoblasts were provided by the UMMS Wellstone Center biobank and have been previously characterized (PMID: 22798623).

Authentication

HEK293 cells were characterized by the manufacturer (ATCC). The following STR profiling was done: Amelogenin: X; CSF1PO: 11,12; D13S317: 12,14; D16S539: 9,13; D5S818: 8,9; D7S820: 11,12; THO1: 7,9.3; TPOX: 11; vWA: 16,19

Muscle cell lines: 15A, 17A sand 18A FSHD human myoblasts and 15V control human myoblasts were provided by the UMMS Wellstone Center for FSHD and have been previously characterized (PMID: 22798623). Based on the referenced literature, authentication of these cells was done by enriching for CD56 positive myogenic cells.

Mycoplasma contamination

HEK293 cells were tested for mycoplasma contamination. No mycolplasma contamination was detected in HEK293 cell lines.

	Muscle cell lines: 15A, 17A sand 18A FSHD human myoblasts and 15V control human myoblasts were not tested for mycoplasma contamination.
Commonly misidentified lines (See ICLAC register)	None of the cells used in this study were listed in ICLAC database of commonly misidentified cell lines.

Animals and other organisms

Policy information about studies involving animals; ARRIVE guidelines recommended for reporting animal research

Laboratory animals	C57BL/6 mice males of 6-9 weeks of age.
Wild animals	No wild animals were used in the study.
Field-collected samples	No field-collected samples were used in the study.
Ethics oversight	Animal studies were performed following the NIH guide for the care and use of laboratories animals.<br>Animal studies were supervised by the Abigail Wexner Research Institute at Nationwide Children's Hospital Institutional Animal Care and Use Committee (IACUC) protocols.<br>This study was granted the IACUC approval number: AR13-00015.

Note that full information on the approval of the study protocol must also be provided in the manuscript.

Human research participants

Policy information about studies involving human research participants

Population characteristics	The following human muscle cell lines were used in this study: 15A FSHD human myoblasts (from a Male proband with 66 years of age), 17A FSHD human myoblasts (from a Male proband with 23 years of age), 18A FSHD human myoblasts (from a Female proband with 36 years of age) and 15V control human myoblasts (from a Female, sister of 15A with 60 years of age). These cell lines were provided by the UMMS Wellstone Center biobank and have been previously characterized (PMID: 22798623).
Recruitment	*Describe how participants were recruited. Outline any potential self-selection bias or other biases that may be present and how these are likely to impact results.*
Ethics oversight	*Identify the organization(s) that approved the study protocol.*

Note that full information on the approval of the study protocol must also be provided in the manuscript.
