# Supplementary material for: Human miRNA miR-675 inhibits DUX4 expression and may be exploited as a potential treatment for Facioscapulohumeral muscular dystrophy
Source: Nat Commun. 2021 Dec 8;12:7128. doi: 10.1038/s41467-021-27430-1 (PMC8654987; doi:10.1038/s41467-021-27430-1)
Supplement: Supplementary file 3 — Description of Additional Supplementary Files [file 41467_2021_27430_MOESM3_ESM.docx]

**Description of Additional Supplementary Files**

File Name: Supplementary Data 1

Description: *miR-675* molecular beacon sequences and *DUX4* target sites.

File Name: Supplementary Data 2:

Description: miRWalk_miRNA targets for *TRIM43* and *KHDC1L* 3’UTR.

File Name: Supplementary Data 3:

Description: miRWalk_miRNA targets for *TRIM43* and *KHDC1L* CDS.

File Name: Supplementary Data 4:

Description: miRWalk_miRNA targets for *DUX4*.

File Name: Supplementary Data 5:

Description: Quantification (percent change) of endogenous *miR-675-5p*, transfected *DUX4* and endogenous *TRIM43* from HEK293 cells treated with the three listed drug regimens, using droplet digital PCR (ddPCR).
